# Identification of microbial metabolic functional guilds from large genomic datasets

**DOI:** 10.3389/fmicb.2023.1197329

**Published:** 2023-06-30

**Authors:** Ryan Reynolds, Sangwon Hyun, Benjamin Tully, Jacob Bien, Naomi M. Levine

**Affiliations:** ^1^Department of Marine and Environmental Biology, University of Southern California, Los Angeles, CA, United States; ^2^Department of Data Sciences and Operations, University of Southern California, Los Angeles, CA, United States; ^3^Wrigley Institute for Environmental Studies, University of Southern California, Los Angeles, CA, United States

**Keywords:** modeling, community assembly, biogeochemical cycling, marine microbiology, microbial metabolisms, functional guilds

## Abstract

Heterotrophic microbes play an important role in the Earth System as key drivers of major biogeochemical cycles. Specifically, the consumption rate of organic matter is set by the interaction between diverse microbial communities and the chemical and physical environment in which they reside. Modeling these dynamics requires reducing the complexity of microbial communities and linking directly with biogeochemical functions. Microbial metabolic functional guilds provide one approach for reducing microbial complexity and incorporating microbial biogeochemical functions into models. However, we lack a way to identify these guilds. In this study, we present a method for defining metabolic functional guilds from annotated genomes, which are derived from both uncultured and cultured organisms. This method utilizes an Aspect Bernoulli (AB) model and was tested on three large genomic datasets with 1,733–3,840 genomes each. Ecologically relevant microbial metabolic functional guilds were identified including guilds related to DMSP degradation, dissimilatory nitrate reduction to ammonia, and motile copiotrophy. This method presents a way to generate hypotheses about functions co-occurring within individual microbes without relying on cultured representatives. Applying the concept of metabolic functional guilds to environmental samples will provide new insight into the role that heterotrophic microbial communities play in setting rates of carbon cycling.

## 1. Introduction

Microbes are the engines that drive many global processes critical for maintaining Earth as a habitable planet, including the cycling of carbon and nitrogen. In particular, heterotrophic microbes (bacteria and archaea) control the rate at which organic compounds are cycled (Pomeroy, 1974; Fuhrman and Azam, 1980, 1982; Falkowski et al., 2008), which has important implications for atmospheric CO_2_ concentrations and thus climate. However, we currently have limited knowledge of what sets the rate of organic matter cycling (Dittmar et al., 2021; Zakem et al., 2021) and how these rates vary as a function of microbial community composition.

Global ecological models, which are used to study large-scale carbon cycling, typically consider the impact of microbial heterotrophy to be a constant or a bulk approximation acting on a generic organic carbon pool (Aumont and Bopp, 2006; Séférian et al., 2013). Thus, these models are unable to capture variations in rates of biogeochemical cycling driven by dynamic and diverse microbial communities. This is partially due to the lack of a tractable framework for explicitly modeling complex heterotrophic microbial communities, their biogeochemical function, and how these functions vary both temporally and spatially. Such a framework requires an understanding of organismal-level metabolic potential (i.e., which metabolic pathways co-occur within individual cells) and how microbes are assembled to form communities. While such a framework exists for phytoplankton (Quere et al., 2005; Raitsos et al., 2008), we lack a similar framework for defining meaningful heterotrophic functional types or metabolic functional guilds. Metabolic functional guilds are defined here as groups of organisms that are capable of the same biogeochemical or ecological function (e.g., nitrogen fixation or chitin degradation) in an ecosystem.

Microbial communities have primarily been characterized using the amplification of marker genes (e.g., 16S small subunit RNA gene). Analysis of functional diversity has either relied upon ‘omics analyses (Venter, 2004; Yooseph et al., 2007; Larkin et al., 2021; Ustick et al., 2021) or closest cultured representatives (Staley et al., 2014; Hornick and Buschmann, 2018; Roth Rosenberg et al., 2021). The former provides an account of which genes are present but does not provide insight into which functions are co-occurring within individual organisms. The latter extends phylogenetic analyses to gain insight into function by using genomic data from the closest cultured representative via tools such as PICRUSt or Tax4Fun2 (Langille et al., 2013; Wemheuer et al., 2020). While this provides insights into the metabolic potential of the community, it relies on having a cultured representative where the vast majority of organisms in the ocean do not have such representatives (Sogin et al., 2006; Parks et al., 2017). In addition, the cultured representative approach relies on the assumption that biogeochemically relevant functions are highly phylogenetically conserved, which may not always hold due to high rates of horizontal gene transfer (McDaniel et al., 2010). Several experimental and observational studies have demonstrated that function and phylogeny are often decoupled in a variety of environments (Louca et al., 2016, 2017, 2018; Tully et al., 2018a). Pangenomics has revealed microdiversity within individual species that results in genetically distinct species sub-groups or sub-clades (Delmont and Eren, 2018) further complicating the link between function and phylogeny.

Recent advances in bioinformatic techniques have allowed for the high throughput assembly of organismal genomes from metagenomes, termed metagenome assembled genomes (MAGs) (Strous et al., 2012; Imelfort et al., 2014; MetaHIT Consortium et al., 2014; Kang et al., 2015, 2019; Lu et al., 2016; Wu et al., 2016; Graham et al., 2017). In addition, microfluidics techniques have enabled the sequencing of single cells [single-cell amplified genomes (SAGs)] (Stepanauskas and Sieracki, 2007; Swan et al., 2011, 2013; Martinez-Garcia et al., 2012; Pachiadaki et al., 2019; Sieracki et al., 2019). Combined, these innovations have led to large datasets of publicly available annotated MAGs and SAGs (Klemetsen et al., 2018; Pachiadaki et al., 2019; Paoli et al., 2021), thus significantly increasing our knowledge of microbial diversity. Most notable is the *Tara* Oceans circumnavigation expedition (Sunagawa et al., 2015), which collected metagenomes from a global set of sampling stations that have been subsequently assembled into thousands of MAGs (Lombard et al., 2014; Baker et al., 2015; Graham et al., 2018; Rawlings et al., 2018; Zhang et al., 2018; Zhou et al., 2019). These large, well-annotated datasets provide an unprecedented opportunity to assess co-occurring functions within a cell for uncultured organisms.

In this study, we present a new statistical approach for defining microbial metabolic functional guilds and show that the guilds we identify are specific and ecologically relevant. This approach also establishes a framework that can be used to generate new hypotheses for co-occurring functions. As our approach is agnostic to phylogeny with no *a priori* phylogenetic data provided, this framework provides an excellent tool for interrogating the metabolic potential of uncultured organisms. This study lays the foundation for defining microbial communities in terms of metabolic functional guilds that will allow us to better understand the role that dynamic microbes play in determining the rates of biogeochemical cycles.

## 2. Materials and methods

### 2.1. Dataset

Three different sources of genomes were used for this analysis, MAGs, isolate genomes (i.e., from cultures), and SAGs. Specifically, we used 1,859 MAGs (Tully et al., 2018b) assembled from the *Tara* Oceans metagenomes (Sunagawa et al., 2015) using the BinSanity v0.2.6.1 technique and assembly pipeline (Graham et al., 2017). Only bins that met the following minimum requirements were assigned as draft genomes and included as MAGs: >90% complete and < 10% contamination, 80–90% complete with < 5% contamination, or 50–80% complete with < 2% contamination. These genomes can be found at NCBI under BioProject ID PRJNA391943. A total of 6,872 SAG genomes were obtained from the GORG-Tropics database (Pachiadaki et al., 2019), which can be found at NCBI under BioProject ID PRJEB33281 and at Open Science Framework under DOI 10.17605/OSF.IO/PCWJ9. Only SAGs with at least 70% completeness were included in our analysis (*N* = 1,733). In addition, 967 isolate genomes and 980 genomes with unresolved provenance (i.e., unclear from the metadata whether MAGs or isolates) were obtained from the MarDB (Klemetsen et al., 2018) (https://mmp.sfb.uit.no/databases/) (accessed 31 May 2018). A composite genomic dataset was generated using the *Tara* Oceans MAGs, isolates, and MarDB genomes (*N* = 3,840). To compare and contrast the guilds derived from different methods of genome reconstruction, two additional datasets were used. The 1,859 known MAGs from the composite dataset were separated out into a second dataset, and the 1,7333 high-quality SAGs from the GORG-Tropics database were separated out into a third dataset.

Genomes from the composite and SAG datasets were classified using the GTDB taxonomy toolkit (GTDB-Tk) (Chaumeil et al., 2022) using r207 of the Genome Taxonomy Database (Parks et al., 2018). GTDB-Tk v2.1.0 utilized Prodigal v2.6.3 (Hyatt et al., 2010) to predict genes on the 3,840 input genomes provided as FASTA nucleotide sequence files. The set of 120 bacterial and 53 archaeal target marker genes used in GTDB-Tk was identified with HMMER 3 v3.1b2 (Eddy, 2011). Phylogenetic estimation was performed with FastTree2 v2.1.11 (Price et al., 2010), and then FastANI v1.32 (Jain et al., 2018) and Mash v2.3 (Ondov et al., 2016) were used to confirm phylogenetic groups with ANI measures. Quality analysis of the genomes in both datasets was performed using CheckM v1.2.1 (Parks et al., 2015). The average completeness for the composite dataset was 90.8% with an average contamination of 1.5%, and the average completeness for the SAG dataset was 80.6% with an average contamination of 0.15%. Phylogenomic trees were constructed for the full set of genomes using GToTree v1.7.05 (Lee, 2019), as well as for the guilds shown in Supplementary Table 2 using the taxonomic classifications from GTDB-Tk to annotate each tree. Similar to GTDB-Tk, GToTree utilized Prodigal v.2.6.3 (Hyatt et al., 2010) to predict functional genes for the 3,840 input genomes provided as FASTA sequence files. Target genes from the pre-built Archaea_and_Bacteria gene set (25 genes) were identified with HMMER 3 v3.3.2 (Eddy, 2011), aligned with muscle v5.1 (Edgar, 2021), trimmed with TrimAl v1.4 (Capella-Gutierrez et al., 2009), and concatenated before phylogenetic estimation was performed using FastTree 2 v2.1.11 (Price et al., 2010).

To further assess the phylogenetic diversity of the composite dataset, we also computed the average nucleotide identity (ANI) and average amino acid identity (AAI). ANI values were computed on the whole genomes using fastANI v1.33 (Jain et al., 2018) while AAI values were computed using fastAAI v0.1.20 (https://github.com/cruizperez/FastAAI). fastAAI also used Pyrodigal (Larralde, 2022), a Python library binding to Prodigal (Hyatt et al., 2010), to predict genes, as well as PyHMMER (Larralde, 2022) to perform the alignments to fastAAI's single-copy protein (SCP) datasets. A full breakdown of this pipeline is presented in Supplementary material S1.

We selected 212 experimentally verified and well-characterized metabolic pathways from the KEGG database (Ogata et al., 1999) (Supplementary Table 1). These functions were chosen due to their biogeochemical (e.g., nitrogen fixation and methanogenesis) and ecological (e.g., motility and chemotaxis) relevance. All genomes were then analyzed using KEGG-Decoder v0.6sbp and KEGG-Expander v0.5 (Graham et al., 2018) to identify the presence or absence of the 212 pathways. KEGG-Decoder is informed by KEGG pathways/modules; however, specific steps and key biogeochemical reactions are broken down to reflect essential steps. Specifically, several different criteria or thresholds were used in order to determine whether pathways were present in a given genome. KEGG-Decoder first assumes that core metabolisms must be present for normal cellular functioning for most organisms, and thus it is unlikely to find a fragmentary pathway that is non-functional. Thus for core metabolisms (e.g., glycolysis, gluconeogenesis, ATP synthase, etc.), a low threshold of 25% total gene presence was used. Conversely, KEGG-Decoder assumes that the same is not true for complex/geochemically relevant pathways, thus a higher threshold is implemented to ensure that it is tracking actual functionality rather than misannotation. Thus, for pathways that were either complex (e.g., multiple branching options), geochemically relevant (e.g., thiosulfate oxidation), or both (e.g., secretion pathways), a total gene presence between 50 and 75% was required. An intermediate threshold of 33–40% total gene presence was used for simple pathways constituting 3 to 4 genes. For “pathways” that possess only a single reaction, presence/absence was directly determined.

This large binary dataset was used as input for metabolic guild identification both using classical methods and our new Aspect Bernoulli (AB)-based method (*see below*). It is important to note that the AB method presented here is not restricted to this number of functions and can be extended to include as many functions or hypothetical proteins as the user desires. Furthermore, genome annotations can be performed in any manner the user desires so long as the resulting data matrix is binary. However, we emphasize that the choice of annotations is paramount in determining the types of metabolic signals the user can receive when running this method. This is a discovery-based dimension reduction method and as such can only directly identify patterns based on the data presented to it.

### 2.2. Classic methods

We tested several clustering and dimensionality reduction methods to attempt to identify microbial metabolic guilds including Non-metric Multidimensional Scaling (NMDS) (Kruskal, 1964) of the functions and complete linkage hierarchical clustering of both the genomes and functions concurrently. NMDS was performed using the *metaMDS* function from the vegan package v2.6.4 (Oksanen et al., 2019) in R v4.2.3 with two dimensions, Bray-Curtis dissimilarity (Bray and Curtis, 1957) and a maximum of 50 iterations. We also analyzed our composite dataset using an agglomerative hierarchical clustering method using the *clustergram* function from the Statistics and Machine Learning toolbox v12.1 from MATLAB R2021a (The Math Works, 2021). We applied these two statistical methods to our composite dataset of 3,840 genomes and assessed their ability to extract a low-dimensional structure of co-occurring functions in the form of guilds.

Finally, we sought a method that could reduce our data to a lower number of dimensions with defined and clear separation into clusters of functions that represent metabolic guilds. Therefore, it was essential that our method could identify signals of metabolic guilds driven by relatively rare functions even in the presence of high abundance functions such as core carbon metabolism or housekeeping genes. This aspect was important because we expected many of these core metabolisms to strongly co-occur due to their essential nature and thus could potentially limit our ability to define more biogeochemically relevant metabolic functional guilds. We found that an augmented AB model was able to best accommodate all of these requirements. We present this model and the underlying statistical method that defines this approach in the following section.

### 2.3. Aspect bernoulli

We used the AB model (Bingham et al., 2009) to perform a statistical matrix decomposition of our binary data matrix *Y*∈*R*^*G* × *F*^. The AB model was selected as it is designed for sparse matrices of binary data. AB is similar to Latent Dirichlet Allocation (LDA) that has been applied to similar problems [e.g., topic modeling, population structure (Pritchard et al., 2000; Blei, 2003)] but is not designed to handle binary data. The AB model assumes that each entry *Y*_*g, f*_ in the data matrix *Y* is a random Bernoulli realization of an underlying scalar probability *V*_*g, f*_∈[0, 1]. Here, *g* denotes genome, and *f* denotes function. In other words, the AB method assumes that the observed pattern in the data is the result of a Bernoulli coin flip based on the probability of a specific function occuring in a specific genome. Thus, we can define another matrix {_*V*_*gf*_}*g* = 1, …, *G, f* = 1, …, *F*_ with the same dimensions as the data matrix that represents these underlying probabilities.

We then assume that this matrix of probabilities {_*V*_*gf*_}*g* = 1, …, *G, f* = 1, …, *F*_ can be defined as the product of two additional matrices β and Γ such that


(1) $$\begin{array}{l} V_{gf}={\text{Γ}}_{g\cdot }\beta_{\cdot f} \end{array}$$

for each probability *V*_*gf*_ in the matrix. The β and Γ matrices are of size G by *k* and *k* by F, respectively, where G is the total number of genomes in the data set and F is the total number of functions. These two matrices allow us to identify *k* groups or aspects in our dataset (see Box 1 for definition). Aspects are distinct from guilds in that they are defined on the entire set of functions, rather than a co-occurring subset of functions (guilds). The term aspect is used to describe the direct output of the AB method. As we describe below, we can then define metabolic functional guilds based on the β matrix, which provides the probability that function *f* is present in a given genome if that genome is associated with the *k*^*th*^ aspect. Particularly, if β_*kf*_ is close to 1 then function *f* is highly associated with aspect *k*. The Γ matrix quantifies how strong the *k*^*th*^ aspect is, within each genome *g*. Specifically, if Γ_*gk*_ is close to 1, then genome g is strongly associated with aspect *k*. and Γ are then optimized using an iterative Expectation Maximization (EM) algorithm as described in Bingham et al. (2009). For a detailed, rigorous description of the methods, please see Supplementary material 1.2.

Box 1 **Terminology Box.**

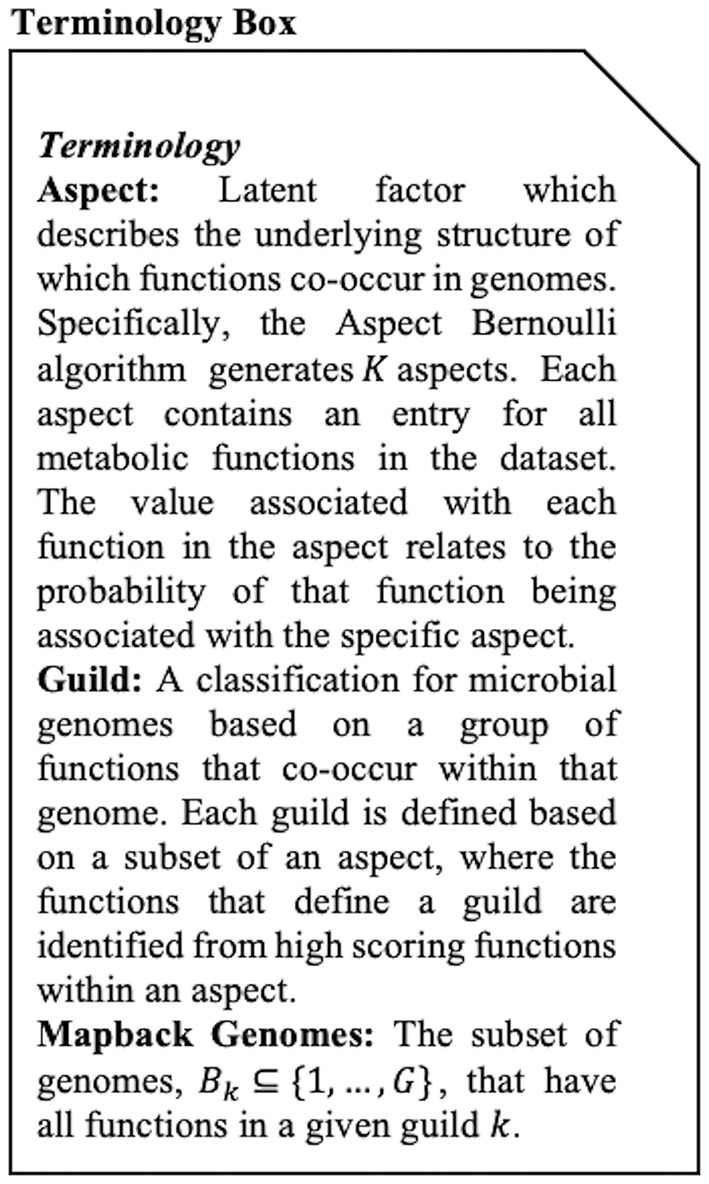

One key advantage of the AB method is that the use of the matrix of probabilities {_*V*_*gf*_}*g* = 1, …, *G, f* = 1, …, *F*_ allows the method to deal with inaccuracies in the data (e.g., false absences or presences) as detailed in the study by Bingham et al. (2009). Specifically, the AB method can accommodate instances where the presence (absence) of a function in the genome is otherwise inconsistent with the main aspects associated with it.

### 2.4. Scoring

In order to define metabolic functional guilds (see Box 1 for definition) from the AB model output, we needed a way to quantify the relative importance of functions within an aspect. To this end, we introduced a post-processing score to order the functions within each aspect such that two conditions were met: (1) functions that were strong indicators of membership in that aspect were highly scored (i.e., if that function was present in a genome, then it was likely that the aspect *k* was present); (2) genomes that were identified as being associated with the aspect *k* were likely to contain functions at the top of aspect *k*'s list (i.e., if genome *g* was associated with the aspect *k*, it was likely to have function A which was at the top of aspect *k*'s list). The functions that combined to define a metabolic functional guild could then be identified based on high-ranking functions in the aspect lists.

To meet the first condition, we posed the following question: having observed a function *f* to be present in a randomly chosen genome *g*, how likely was it that the function was present due to aspect *k*? We could quantify this likelihood by calculating


(2) $$\begin{array}{l} r_{fk}=\frac{1}{G}\overset{G}{\underset{g=1}{\sum }}P\left(Z_{gfk}=1\;|\;Y_{gf}=1\right). \end{array}$$

Using Bayes' rule, we computed the above conditional probability in terms of the AB parameters:


(3) $$\begin{array}{l} P\left(Z_{gfk}=1|Y_{gf}=1\right)=\frac{\Gamma_{gk}{\;\beta }_{kf}}{V_{gf}}. \end{array}$$

Next, we identified the genomes that were most strongly associated with each aspect (i.e., having large Γ values). We will hereafter refer to this set of genomes *A*_*k*_⊆{1, …, *G*} as aspect *k*'s “probabilistic representatives.” We filtered {1, ⋯ , *G*} into *K* non-overlapping sets *A*_1_, ⋯ , *A*_*K*_, each set *A*_*k*_ was defined as the genome *g* that placed the highest value of Γ_*g*_ on *k* and also had a large enough Γ_*g, k*_ = *P*(*Z*_*gfk*_ = 1) (specifically, Γ_*g, k*_>2/*K*). This 2/*K* threshold ensured that we excluded genomes that had nearly uniform Γ vectors. For our composite dataset, this threshold did not exclude any genomes.

From *A*_*k*_, we calculated *q*_*fk*_:


(4) $$\begin{array}{l} q_{fk}=\frac{\sum_{g\in A_{k}}Y_{gf}}{\frac{1}{F}\sum_{f=1}^{F}\sum_{g\in A_{k}}Y_{gf}} \end{array}$$

which is the ratio of the abundance of each function within *A*_*k*_ and the mean abundance within *A*_*k*_. Finally, we multiplied the marginal probability *r*_*fk*_ (Equation 1) by the adjustment factor *q*_*fk*_ (Equation 4). This gave us the score metric *s*_*fk*_ that we used to identify our guilds:


(5) $$\begin{array}{l} s_{fk}=r_{fk}\cdot q_{fk} \end{array}$$

**Figure 1:**
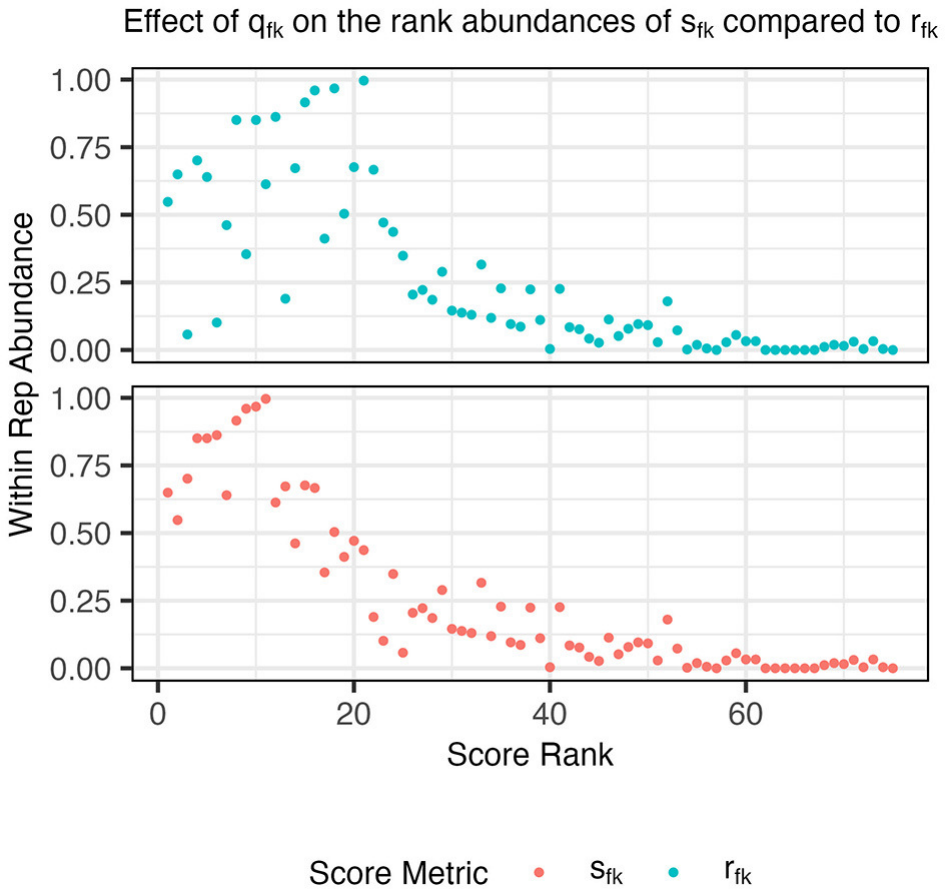
Abundances of functions within an example aspect's probabilistic representatives, A_k_, compared to their score rank before (*r*_*fk*_, cyan) and after (*s*_*fk*_, orange) applying the score adjustment *q*_*fk*_ (step 2). After the adjustment, a large density of points in the upper left quadrant is observed indicating that the highest rank functions using *s*_*fk*_ are also found within a large number of probabilistic representative genomes.

In this score, *q*_*fk*_ upweights functions *f* that are more abundant among probabilistic representatives of aspect *k* than average (Figure 1) and makes the score (Equation 5) more comparable across aspects. Since a function that is highly specific to aspect *k* is highly scored, top-scoring functions are attractive candidates for forming metabolic function guilds from aspects. Next, we describe how to choose a small set of functions to form such guilds. The full algorithm for the AB procedure can be found in the extended methods (Supplementary material S1).

### 2.5. Guild identification and mapback genomes

After identifying the probabilistic representatives *A*_*k*_ based on our pipeline, we further narrowed each aspect down to metabolic functional guilds *F*_*k*_ according to the scores *s*_*fk*_. Then, we obtained the mapback genomes *B*_*k*_ (see Box 1) for guild *F*_*k*_ as the set of genomes possessing all of the functions in *F*_*k*_. We used two alternative approaches to identify the set of functions that comprise metabolic functional guilds: (1) using a fixed number of functions, five functions in this case (Option 1 in Supplementary material S1) or (2) requiring a minimum number of genomes in the dataset to be associated with a given guild (Option 2 in Supplementary material S1). The number of mapback genomes is an important criterion in our pipeline, as it quantifies how strongly the original data support the proposed metabolic functional guilds. For instance, if we found many mapback genomes for a fixed-size functional guild, we would be more confident in the validity of that guild.

### 2.6. Guild specificity

A key objective of the pipeline was to identify functions co-occurring within individual genomes that were meaningfully associated. Ideally, for a guild *k* containing functions A and B, the presence of function A in a genome would indicate both that the genome was a member of guild *k* and that the genome would also contain function B. To test the association between pairs of functions within our guilds, we calculated the confidence (Agrawal et al., 1993) of seeing B given A (*A*→*B*) as


(6) $$\begin{array}{l} Conf(A,B)=\frac{\sum_{g=1}^{G}Y_{gA}Y_{gB}}{\sum_{g=1}^{G}Y_{gA}} \end{array}$$

where A and B are functions from our dataset and *Y*_*gA*_ and *Y*_*gB*_ are the presence or absence of A and B in genome *g*. High confidence values suggested that the presence of function B was highly conserved with that of function A. We computed the forward and reverse confidence values for every pair of functions in the guilds identified from our data. Because of the way we defined mapback genomes, these confidence values were all 1 within our mapback genomes and ranged between 0 and 1 for our ‘outgroup' genomes (i.e., the rest of the dataset).

### 2.7. Artificial datasets

The number of aspects, *K*, is a free parameter in the AB model that determines the maximum number of guilds that can be identified. The ideal choice of *K* is dataset specific and is a function of the underlying structure of the data matrix. To test the impact of this choice on the resulting guilds identified by our method, we constructed a large collection of synthetic datasets comprised of either one or three artificial guilds appended to our original composite dataset of 3,840 genomes and 212 functions. These guilds were defined to be “perfect” guilds when genomes either had all the artificial guild functions or none of them. For example, an artificial guild with 5 functions and 2% total abundance in the dataset would have all 5 functions perfectly co-occurring in 77 genomes, while the remaining 3,763 genomes would not possess any of these artificial functions (all zeros). Guild parameters were drawn from three possible abundances (2%, 5%, or 10% of the genomes containing the artificial guild) and three possible sizes (guilds consisting of 5, 7, or 9 functions) with all unique combinations tested (Supplementary Table 2). Each artificial guild was inserted in a non-overlapping manner such that each genome could only belong to a maximum of one artificial guild. For each combination, we created 100 replicates of our synthetic data. Additional sensitivity analyses were conducted where we assigned guilds randomly, allowing some genomes to belong to multiple artificial guilds (Supplementary material S2).

### 2.8. Data visualization

All data visualizations in MATLAB were performed using the Statistics and Machine Learning Toolbox v12.1 from MATLAB R2021a (The Math Works, 2021). Data visualizations in R v4.2.3 were performed using the ggplot2 v3.4.2 and ggbreak v0.1.1 packages (Wickham, 2009; Xu et al., 2021), as well as the lattice v0.21.8 package (Sarkar, 2008).

## 3. Results

### 3.1. Phylogeny of datasets

The phylogeny of our composite dataset of 3,840 genomes was assessed using GtoTree and GTDB-Tk. From this large dataset, 65 genomes (60 archaeal and 5 bacterial) were excluded due to insufficient marker gene coverage. Another 39 genomes that were included in the tree were flagged during the quality assessment step for high redundancy estimates (an average of 16.7% redundancy) but were still highly complete (an average of 95.7% completeness). Of the 3,775 high-quality genomes, there were 3,529 bacterial genomes representing 51 unique bacterial phyla. Among these phyla were the key marine superphylum Proteobacteria (Yarza et al., 2014) with 1,774 genomic representatives, as well as other notable phyla such as the Cyanobacteria (108 genomes), Bacteroidota (545 genomes), Firmicutes (111 genomes), Desulfobacterota (55 genomes), and the Verrucomicrobiota (91 genomes). In addition, there were 246 archaeal genomes representing 2 unique archaeal phyla, Thermoplasmatota and Thermoproteota. Supplementary Figure 1 shows the full phylogenomic tree visualized in the iTOL web application (Letunic and Bork, 2021), which is colored by individual bacterial phylum identity.

We passed our high-quality SAG dataset of 1,733 genomes through GtoTree and GTDB-Tk and determined the phylogeny for 1,415 genomes (Supplementary Figure 2). In total, 318 genomes (301 bacterial and 17 archaeal) were excluded for insufficient marker gene coverage while three of the included genomes were flagged during the quality assessment step for high redundancy estimates (an average of 14% redundancy). Of the 1,415 high-quality genomes, there were 1,409 bacterial genomes representing 9 unique bacterial phyla and 6 archaeal genomes representing 2 unique archaeal phyla. Like the composite dataset, many of the bacterial genomes were classified in the phylum Proteobacteria (1,158 genomes). The next two largest phyla were Bacteroidota (103) and Cyanobacteria (83). Collectively, these three phyla accounted for 95.4% of all SAGs with an ascribed bacterial phylogeny.

### 3.2. Classic methods

**Figure 2:**
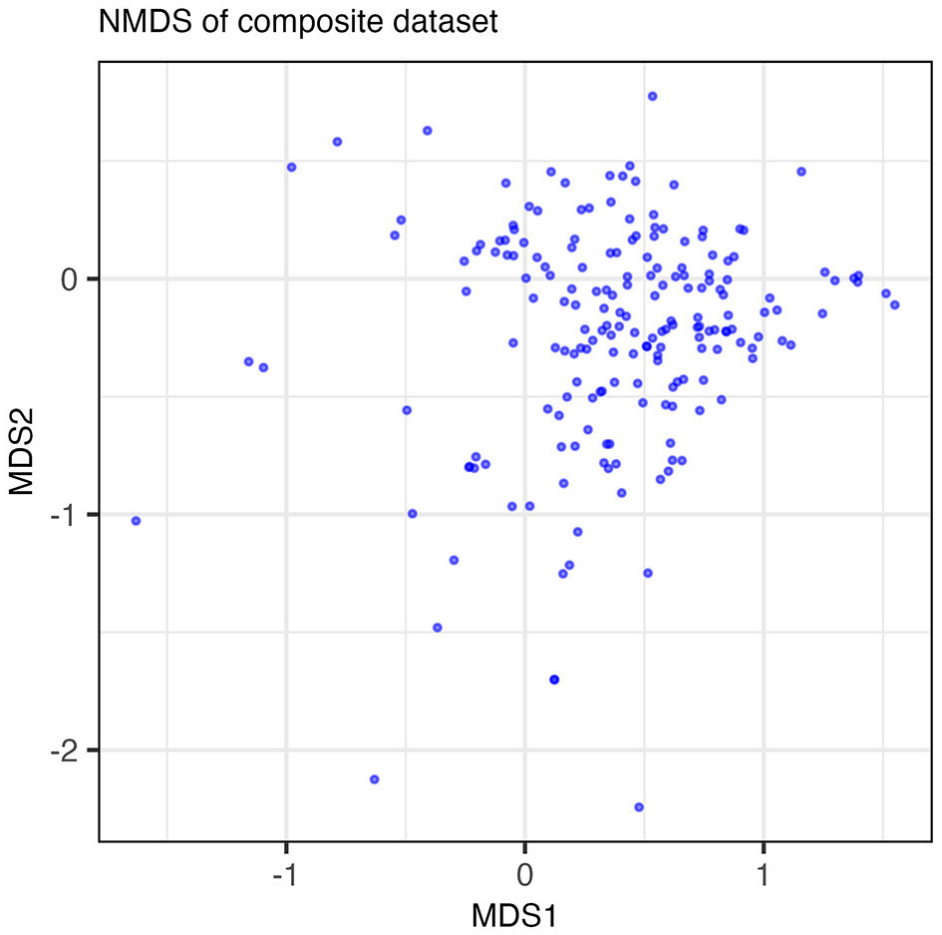
Results of the NMDS run on the composite dataset. Points plotted are the loadings of the functions in the dataset on MDS axes 1 and 2. Points are semi-transparent to emphasize points that overlap one another. The NMDS algorithm did not reach convergence with a minimum stress value of 0.211.

We applied two classic statistical methods (NMDS and *clustergram*) to our dataset and assessed their ability to extract the low-dimensional structure of co-occurring functions in the form of guilds. The results of the NMDS are shown in Figure 2 where each point in the NMDS represents a function such that clusters of points could, potentially, indicate guilds. No distinct features emerge along either axis. The majority of data points group into a dense cloud of points with no clear separation along an axis of variance. While approaches for analyzing variance in reduced dimensions, such as NMDS, can be powerful for identifying clusters of similarly acting samples, NMDS was unable to identify clusters that could be interpreted as metabolic guilds when applied to our dataset.

**Figure 3:**
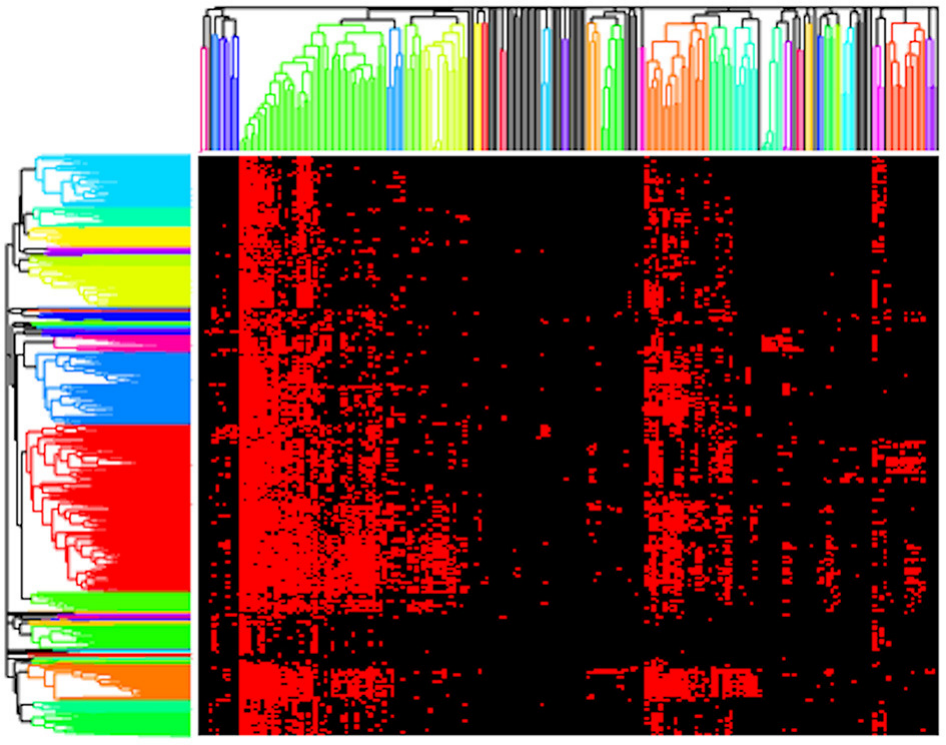
Resulting clustergram plot on the presence/absence pathway data for our composite dataset (red = present, black = absent) using a cut height of 0.9 with rows (genomes) and columns (functions) clustered based on Jaccard distance.

Next, we present results using a standard clustering approach, namely hierarchical clustering, as implemented by *clustergram*. Here, we clustered both the genomes and functions (rows and columns) using the Jaccard distance metric with complete linkage and two different cut heights, 0.9 and 1 (Figure 3). We selected the Jaccard distance for *clustergram* because of the binary format of our data. However, unlike the AB method, Jaccard treats all presences/absences equally and thus does not provide differential weights for rare vs. highly abundant functions. We chose to use cut heights of 0.9 and 1 based on the resulting dendrograms as they produced clusters among both rare and high abundance functions. At lower cut heights, we found that a large bulk of the functions clustered out as singletons, and the clusters that formed were primarily the core, high abundance functions. Thus, we considered that 0.9 and 1 were good values for comparing the microbial metabolic functional guilds identified by *clustergram* and AB.

Applying *clustergram* to our data with a cut height of 0.9 yielded 30 distinct clusters of functions that we interpreted as potential metabolic guilds (Figure 3). These clusters averaged 5.8 functions (ranging from 2 to 42 functions) and 38.8 mapback genomes (ranging from 3 to 354 genomes). Approximately 20% of the total functions (*N* = 42) were in a single guild of highly abundant core functions. We also tested *clustergram* with a cut height of 1 that produced 17 distinct clusters of functions. The average number of functions in a cluster increased to an average value of 11.1 (ranging from 2 to 66 functions) but the number of mapback genomes dropped sharply to an average of just 3.2 mapback genomes (ranging from 0 to 17 genomes) per guild. Seven of these guilds had no mapback genomes, and the two largest guilds alone accounted for 46.7% of the total data used for this clustering procedure.

We identified several disadvantages of the classic statistical methods. First, large numbers of core metabolisms found in many genomes (such as housekeeping genes, core carbon metabolism, etc.) formed huge guilds with few mapback genomes, which were therefore not informative as metabolic guilds (see Figure 3). Second, these methods do not permit functions to be part of more than one guild, which is inconsistent with the high functional redundancy that has been demonstrated in microbial communities (Louca et al., 2016, 2017, 2018; Tully et al., 2018a). Finally, these methods do not provide an intrinsic ranking of the importance of each function for defining a guild, e.g., the functions that are strong indicators of membership in the guild. In the following section, we will compare the guilds from *clustergram* to that of the AB model and demonstrate that both methods identify similar guilds but that *clustergram* both breaks the AB guilds up into smaller groups (fewer functions) and results in guilds with fewer mapback genomes. Thus, the AB method can better capture metabolic functional guilds that contain a meaningful number of functions (>3) with substantial numbers of mapback genomes.

### 3.3. AB model

In the following sections, we present an assessment of the robustness of the AB model for detecting guilds, a summary of the AB model guilds from the composite dataset, and then a comparison between the AB model and the classic methods.

#### 3.3.1. Choosing a value for K

The AB model requires the user to define *K* prior to running the algorithm. To test the impact of the choice of *K* on the ability to detect different-sized guilds (i.e., numbers of functions) and guilds with different abundances in the dataset (i.e., frequency), we ran the artificial datasets through the method with a wide range of *K* values (*K* = 5, ⋯ , 20). This analysis (described in Supplementary material S2 and summarized below) identified a clear trade-off between using low *K* values, which inhibited the detection of low abundance guilds, and using high *K* values, which overfitted the dataset. The values that qualify as “low” vs. “high” *K* values will be specific to the dataset. The analysis described below allows the user to identify a range of reasonable *K* values for a given dataset and the type of guilds (e.g., abundance and size) that are being targeted in the analysis. For this study, we manually assessed guilds derived from *K* values within the identified range in order to select our final value of *K* (*K* = 10). We recommend that a similar analysis be performed prior to applying this method to a new dataset.

We quantified the ability of our method to identify artificial guilds in our artificial datasets (see Section 2) over a range of *K* values using two metrics: hit rate and extra hits. The hit rate describes the overall frequency with which we identified our artificial guilds. In the ideal case, we would observe all of an artificial guild's functions present at the top of the score-ordered function list (top 15) in exactly one aspect. Thus, for a simulation using three distinct artificial guilds, we would expect to see three hits per simulated dataset (i.e., each guild showing up at the top of only one aspect list), which would give us a 100% hit rate, or a hit rate frequency of 1. Extra hits catalog instances where we observed an artificial guild occurring at the top of more than one aspect list, i.e., an artificial guild being divided across two aspects.

**Figure 4:**
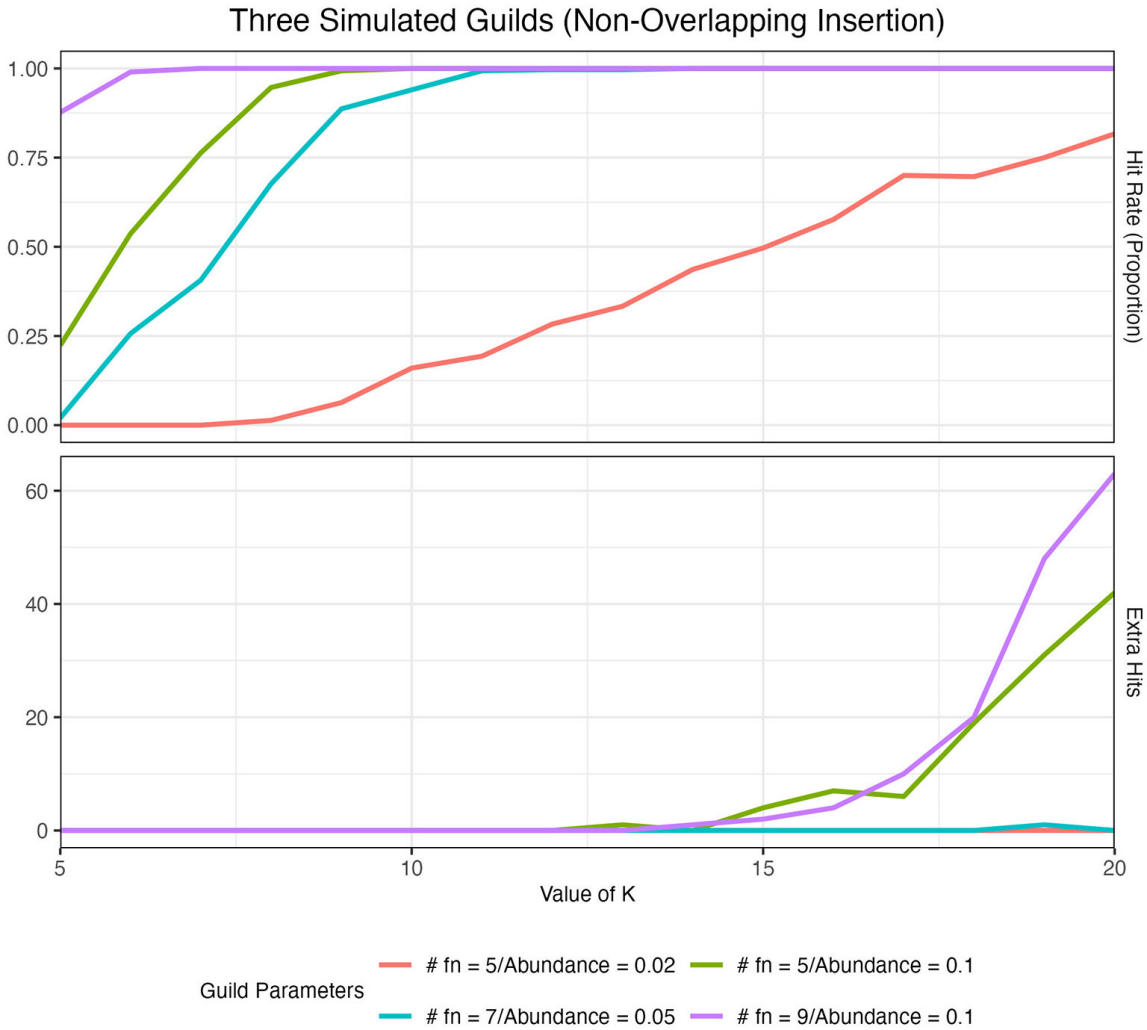
Hit rate and the number of extra hits for 100 simulated datasets with three artificial guilds inserted in a non-overlapping manner across a range of K values. Results are colored by the guild parameters where #fn denotes the number of functions in each artificial guild. The red (#fn = 5/Abundance = 0.02) vs. the green (#fn = 5/Abundance = 0.1) lines illustrate the impact of a change in guild abundance. The impact of guild size on hit rate and extra hits is shown in Supplementary Table 2.

The size of the guild and abundance of the guild in the dataset impacted the ability of the method to identify artificial guilds at different *K* values (Figure 4). As guild size and abundance in the dataset increased, the hit rate at low *K* values increased to 1. In other words, it was easier to identify larger and more abundant guilds, as one might expect. When *K* was low, extra hits were zero. As we increased the value of *K*, the hit rate remained high, but we started to see extra hits. When guilds were large and/or abundant, extra hits increased more quickly and at lower values of *K* than for smaller and less abundant guilds. This analysis demonstrated that when the choice of *K* was too small, only the largest and most abundant guilds were identified (under-fitting system). On the other hand, if *K* was too large, guilds showed up in multiple aspects (over-fitting system). We concluded that a good range for *K* was around the point where the hit rate was maximized while extra hits remained zero. A full analysis of the impact of guild size, guild abundance, and *K* value on guild identification, as well as the impact of randomly inserting guilds and the number of artificial guilds inserted, is presented in Supplementary material S2.

We also tested various numbers of iterations for the expectation-maximization (EM) algorithm implemented as detailed by Bingham et al. (2009) to determine how quickly the model converged to a local maximum. For each iteration value (ranging from 10 to 1,500 steps), we initialized and ran 10 random restarts. For our chosen value of *K* = 10, the likelihood appeared to plateau at its maximum value after ~500 iterations (Supplementary Figure 12). We also assessed the stability of the AB results and showed that the identification of guilds was consistent across runs initialized with different random seeds (Supplementary Figure 13).

#### 3.3.2. Guild identification in the composite dataset

**Figure 5:**
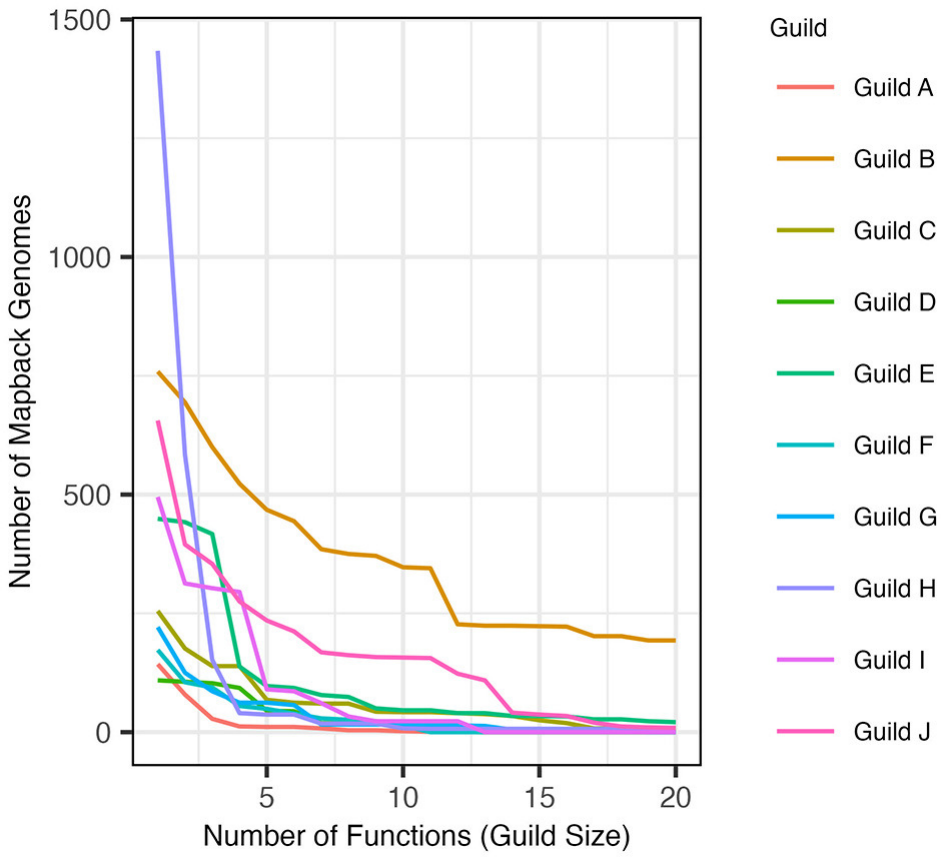
The number of genomes that possess all of the functions in a guild (mapback genomes) as guild size is expanded to include more functions in decreasing score order (starting at size 2).

The AB method successfully identified guilds within the composite dataset that were found in a substantial number of genomes in the dataset and contained functions that were specific to that guild (see Section 2). When defined using the top 5 scoring functions (approach 1), the resulting guilds averaged 116.2 mapback genomes (ranging from 11 to 468 genomes). When guilds were defined to include functions co-occurring within at least 100 genomes (approach 2), the average guild size was 5.7 functions per guild (ranging from 2 to 20 functions). Figure 5 shows the number of mapback genomes present in the dataset as the number of functions defining each guild is increased from 2 to 20.

**Figure 6:**
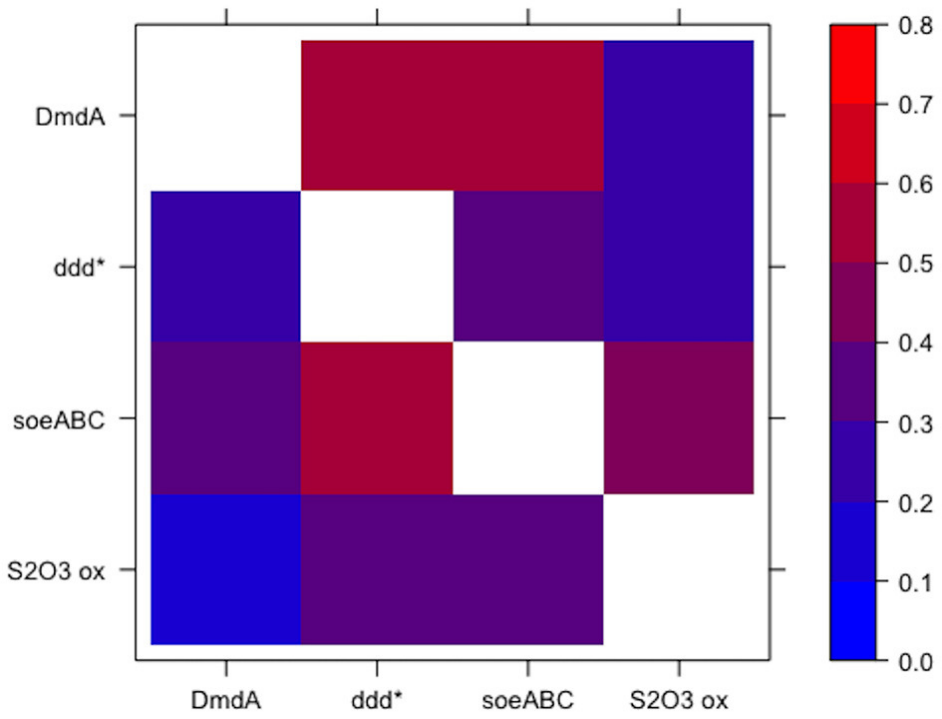
Specificity of guild function pairs for a guild related to the degradation of DMSP. Values are shown for the confidence of the guild function pairs in the outgroup genomes such that low values indicate high specificity of the guild function pairs for the DMSP guild. Note that the colorbar is scaled from 0 to 0.8. The diagonal is omitted since it is 1 by definition. The axes are non-symmetric because DmdA → ddd* is fundamentally different from ddd* → DmdA (see Equation 6).

Both approaches for defining guilds resulted in guilds comprised of functions that were specific to that guild. When looking at the co-occurrence of each pair of functions from the guild set of functions (guild function pairs), low confidence values were observed in the outgroup genomes for each guild function pair as compared to the value of 1 for the guild function pairs in the mapback genomes (by definition). Guilds identified using approach 1 (top 5 scoring functions) had a 0.455 average confidence value in the outgroup genomes. However, many pairs of functions were substantially less conserved in the outgroup genomes (i.e., these pairs were strongly indicative of membership in the guild). For this, we looked at the minimum outgroup confidence value across all pairs of functions in each guild (i.e., the two functions that most strongly indicated membership in the guild). For approach 1, the average across all 10 guilds (*K* = 10) of the minimum confidence values was 0.09 (ranging from 0.029 to 0.132). In other words, functions *A* and *B* in guild *k* were found together only ~10% of the time in the non-mapback genomes and 100% of the time in the mapback genomes. Guilds defined using approach 2 (~100 mapback genomes) had a 0.338 average confidence value in the outgroup genomes and a 0.029 (ranging from 0 to 0.105) average minimum confidence value. Figure 6 shows an example heatmap of both the forward and reverse confidence values for a putative DMSP guild. Low confidence values for the outgroup genomes confirm that this method identified functional co-occurrences that are specific only to a subset of genomes.

### 3.4. Comparison between the AB model and clustergram guilds

**Figure 7:**
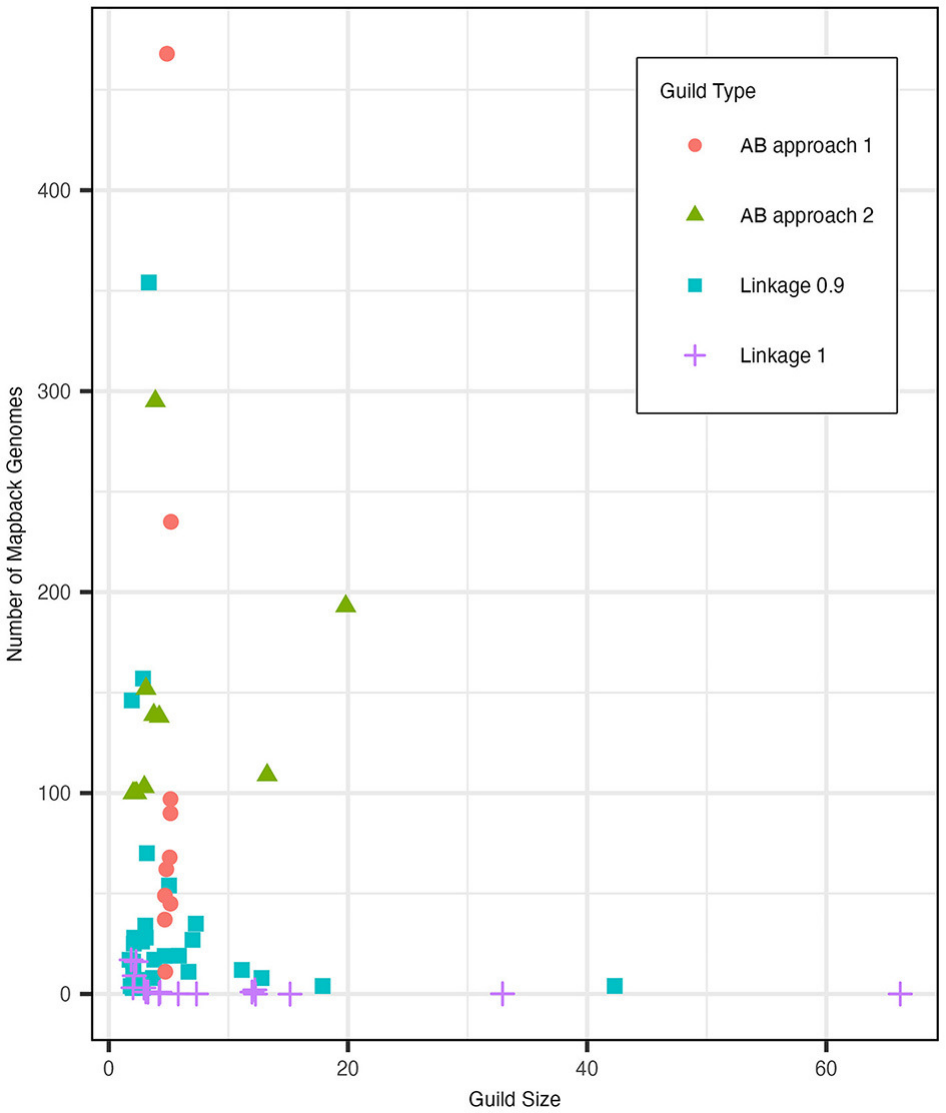
The distribution of guild sizes (number of functions) and the number of mapback genomes for guilds generated with clustergram at cut heights of 0.9 (blue square) and 1 (purple plus sign) as well as for AB. AB approach 1 (red circle) defined guilds using a fixed size of 5 functions while AB approach 2 (green triangle) defined guilds using a minimum mapback genome cut-off of 100. Points were jittered using the built-in position_jitter function in the ggplot2 package v3.4.2 with h = 0.1, w = 0.35 using the random seed 123.

We compared the guild sizes and mapback genome numbers of the *clustergram* guilds to guilds generated using the AB method approaches 1 and 2. Figure 7 shows the distribution of guild sizes vs. the number of mapback genomes for each of these three methods. Based on our simulated data analysis described in Section 3.3, we determined that *K* = 10 was an appropriate number of guilds for the AB method. Overall, we found that the *clustergram* method identified more guilds with fewer functions and fewer mapback genomes than the AB method. Specifically, with a cut height of 0.9, *clustergram* identified three times as many guilds (*N* = 30) as the AB method (*N* = 10). Of these 30 *clustergram* guilds, the majority (60% of the guilds) possessed three or fewer functions with 33.3% of the guilds constituting just a pair of functions. When we used the conservative criteria of at least 100 mapback genomes per guild (approach 2), the AB method generated a comparable number of guilds with 3 or fewer functions (50% of the total guilds). However, the two methods differ substantially in terms of number of mapback genomes identified for each guild. *Clustergram* yielded guilds with an average of 38.8 mapback genomes per guild, substantially less than the two AB methods which averaged 116.2 and 142.9 mapback genomes for approaches 1 and 2, respectively. When we reduced the threshold for AB approach 2 to the *clustergram* average of 39 mapback genomes per guild, we found just one guild with three or fewer functions (10% of the total guilds). To make a more direct comparison to the *clustergram* guilds, we re-ran the AB pipeline with *K* = 30. Allowing for a higher number of guilds in the AB method resulted in a similar number of mapback genomes per guild as the runs with K = 10 with an average of 113 mapback genomes (ranging from 0 to 1436) for approach 1 and with only one guild having no mapbacks. However, when *K* = 30, the AB method resulted in a high frequency of duplicate guilds, either fully duplicated or partially duplicated (see Figure 4 and Section 3.3.1).

To test the impact of the cut height on guild size, we increased the *clustergram* cut height to 1 (Supplementary Figure 8). This results in a more similar number of total guilds (17 for *clustergram* compared to 10 for AB) between the different methods. A cut height of 1 reduced the number of small *clustergram* guilds (3 or fewer functions) to 41.2%. However, this even further decreased the number of mapback genomes for each guild (an average of 3.2 genomes per guild with some guilds having no mapbacks). For both cut heights, *clustergram* identified one guild with 42 functions (cut height = 0.9) and 66 functions (cut height = 1), which correspond to 19.8% and 31.1% of all functions in the dataset, respectively. This large guild was comprised entirely of highly abundant functions and was substantially larger than the largest guild produced by AB approach 2 (28 functions using the lower threshold of 39 or more mapback genomes). Furthermore, the large *clustergram* guild had just 4 and 0 mapback genomes for cut heights of 0.9 and 1, respectively, while the 28-function AB guild had 61 mapback genomes. Finally, we attempted using a dynamic cut height method for clustering functions which improved the guild sizes and number of mapback genomes over the static height but still resulted in guilds with fewer mapback genomes than the AB guilds (see Supplementary material 1.3).

We next assessed the differences in guilds functions identified by the two methods using AB approach 1 where guilds were defined with a static number of functions. We observed several reoccurring patterns. When using a cut height of 0.9 for *clustergram*, the five AB guild functions were typically split between two distinct *clustergram* guilds (range split between 1 and 3 guilds) with only two of the ten AB guilds being contained within a single *clustergram* cluster. When we examined the *clustergram* guilds that contain the AB guild functions, we found that they average 52.8 mapback genomes compared to 116.2 for the corresponding AB guilds. This suggests that the AB method can identify groups of functions that are more commonly found together in the dataset.

Increasing the cut height to 1 resulted in fewer *clustergram* clusters and marginally reduced the fragmentation of AB guilds between *clustergram* guilds with AB guilds now being split across 1.7 *clustergram* guilds on average (ranging from 1 to 3 guilds). At this linkage, the *clustergram* guilds which contained the AB guild functions had on average 30 additional functions (ranging from 5.5 to 61) and only 0.33 mapback genomes (ranging from 0 to 1) compared to the corresponding AB guilds which had 116.2 mapback genomes (ranging from 11 to 468). There were several instances (4 of 10), where the AB guild functions clustered fully or partially into the large *clustergram* guild with 66 functions containing the highly abundant functions in the dataset with no mapback genomes.

This analysis demonstrated that both the AB and clustering methods can identify functional guilds from our dataset and that there was an overlap in the functions that were grouped together into guilds using the two methods. We showed that the AB guilds both contained more functions and were more highly represented in the dataset (have substantially more mapback genomes) than the guilds defined using the clustering method. As with any method, there are both advantages and disadvantages to the AB method. One disadvantage of the AB method is the need to choose a value of the free parameter *K*, which determines the number of guilds identified (see discussion above in Section 3.3.1). However, we demonstrate how a user can use our pipeline to make an informed decision as to how to choose the best value of *K*. Another key distinction between the two methods is that clustering methods precisely define the functions belonging to each guild. The AB method provides information both about which functions are strong indicators of the guild and which genomes have a high probability of membership in the guild. The user must then decide which set of functions to define as a guild. We provide two approaches for making this distinction and highlight how this additional information generated by the AB method can be used to generate hypotheses (see discussion below in Section 4.1). Additional advantages to the AB method are that the AB method does not require all functions to be members of a guild or a function to be a member of just one guild and that the AB method can distinguish between false and true absences/presences in the dataset. Finally, it is important to note for the AB method that if there are mapback genomes for a guild then the guild is by definition meaningful (i.e., found in the dataset). However, the absence of a guild does not necessitate that that guild does not exist. The AB method might not have identified a guild for several other reasons, including other structures in the data matrix which can make rare guilds difficult to find, or the absence of a key annotation that is crucial for distinguishing it from the rest of the dataset.

## 4. Discussion

### 4.1. Emergent microbial metabolic guilds

Our approach identified several biogeochemically relevant metabolic functional guilds with numerous genome representatives in the composite dataset. It is important to note that these guilds emerged from this analysis without any curation or *a priori* knowledge. As such, the identification of known guilds (e.g., photosynthesis) is a strong indication that the method can detect biologically meaningful phenomena even when these associations are in low abundance in the dataset. In this study, we highlight three emergent guilds and draw connections to previously identified co-occurring biochemical processes. The other seven guilds identified by the method are also of significance (11–235 mapback genomes) and are listed in Supplementary Table 4. For example, we identified a guild associated with phosphorus acquisition (C-P lyase genes, see Section 4.2) and several associated with different types of carbon metabolisms (see Guilds 8 and 9 in Supplementary Figure 4). However, for succinctness, we describe in detail just three guilds that illustrate the power of the AB method.

The photosynthetic functions served as a good test case of our method. Our composite dataset was curated in such a way that photosystems I and II were only present in 2.5% (*N* = 95) and 2.7% (*N* = 105) of the genomes, respectively. However, our method was able to identify a photosynthesis guild with 10 total functions including photosystems I and II, NAD(P)H quinone oxidoreductase, cytochrome *b*_6_*f* complex, and RuBisCO (Supplementary Table 4). This 10-function guild had 12 mapback genomes in the composite dataset. We were also able to identify this photosynthetic guild in the SAG dataset where photosystems I and II have abundances of 6.3% and 5.8%, respectively. The identification of this well-characterized system provided an excellent “ground truth” validation of our method.

The approach identified a guild related to the consumption of the organic sulfur compound dimethylsulfoniopropionate (DMSP). This guild consisted of DMSP demethylation, DMSP lyase, and sulfite dehydrogenase (quinone), and had 139 mapback genomes. These three functions were the highest-ranked functions within a single aspect (Table 1). For this analysis, we assessed the presence of at least one of 7 different DMSP lyases (DddL, DddQ, DddP, DddD, DddK, DddY, and DddW). DMSP lyase has been shown experimentally to co-occur with the enzyme DMSP demethylase (DmdA), which performs the demethylation reaction for DMSP (Reisch et al., 2008, 2011), though this association is not obligatory. These pathways have been characterized in abundant marine clades, such as Roseobacters (Moran et al., 2007) and SAR11 (Tripp et al., 2008). Sulfite dehydrogenase has also been implicated as a potential pathway through which DMSP-derived sulfur is reduced from sulfite to sulfate (Reisch et al., 2011).

**Table 1:** Top 15 functions based on score (see Section 2) for two aspects related to DMSP degradation and motility.

**DMSP aspect**	**Scores**	**Motility aspect**	**Scores**
**DMSP demethylation**	30.908	**Type II Secretion**	20.603
**DMSP lyase (dddLQPDKW)**	29.901	**Ubiquinol Cytochrome c reductase**	18.733
**Sulfite dehydrogenase(quinone)**	27.231	**Cytochrome-c oxidase cbb3-type**	17.174
Trimethylamine methyltransferase	22.441	**Flagellum**	12.752
Dimethylamine/trimethylamine dehydrogenase	17.902	**Phospholipid SBP**	12.180
Putative simple sugar SBP	16.735	**Chemotaxis**	11.285
Microcinc SBP	13.544	**Glyoxylate shunt**	7.971
Ubiquinol cytochrome c reductase	13.391	Thiamin biosynthesis	7.577
Taurine SBP	13.029	Phosphate transporter	7.430
Glycine betaine/proline SBP	12.989	Cytochrome bd complex	7.406
General l-amino acid SBP	12.160	Type I Secretion	7.304
Spermindine/putrescine SBP	11.625	Cationic peptide SBP	7.006
Putative spermidine/putrescine SBP	11.493	Ammonia transporter	6.610
Tungstate SBP	10.723	Sec/SRP	6.484
***Thiosulfate oxidation***	10.663	TCA cycle	6.458

Functions that constitute the resulting DMSP and motility guilds are highlighted in bold and bold and italics, respectively. SBP is the substrate-binding protein associated with the respective ABC transporter.

The AB method suggests that there are several additional functions that might commonly co-occur with these three DMSP-related functions (Table 1). For example, taurine and glycine betaine transport, either into the cell to meet metabolic demands or out of the cell to excrete waste products, could be features of this guild. In fact, previous work suggests that many Roseobacters utilize a diverse suite of labile dissolved organic sulfur (DOS) metabolites to meet their sulfur requirements (Landa et al., 2019). In a co-culture experiment with *R. pomeroyi* strain DSS-3 and two phytoplankton species, Landa et al. (2019) demonstrated enriched expression patterns of transport and catabolism genes for seven sulfur-rich phytoplankton exometabolites, including DMSP and taurine. These findings are consistent with the fact that both DMSP and taurine are produced in high concentrations by certain phytoplankton groups (Saltzman and Cooper, 1989; Jackson et al., 1992). The nitrogen-rich compatible solute glycine betaine is also produced by certain phytoplankton groups (Keller et al., 1999) and has been implicated as a nitrogen source for Roseobacters (Moran et al., 2007). Therefore, the capacity to use these substrates co-occurring within a single organism is consistent with known ecological interactions and might indicate that organisms in the DMSP guild could be associated with the phycosphere. Including taurine as a 4^th^ function in the guild resulted in 100 mapback genomes, including glycine betaine as a 4^th^ function resulted in 134 mapback genomes, and including both (5 function guild) resulted in 98 mapback genomes.

Thiosulfate oxidation also occurs in the top 15 ranked score list (rank 15). Previous experimental study has shown that this pathway is involved in DMSP degradation (Reisch et al., 2011). In fact, if we included thiosulfate oxidation within the DMSP guild, we obtained a guild of four DMSP functions with 89 mapback genomes in the composite dataset all co-occurring with a high degree of specificity (Figure 6).

The last example guild was a large guild related to motile microbial lifestyles. The key functions in the motility guild were type II secretion, *cbb*_3_-type cytochrome *c* oxidase, flagellum, chemotaxis, ubiquinol cytochrome *c* reductase, a phospholipid SBP, and the glyoxylate shunt, totaling seven guild functions with 385 mapback genomes (Table 1). These functions are all consistent with copiotrophic lifestyles where organisms are motile and capable of responding to signals in the environment through chemotaxis. Similar to the DMSP guild, a key advantage to our approach is that it provides a list of functions that co-occur with classic “copiotrophic” functions (e.g., chemotaxis and flagellum) with high specificity to the guild mapback genomes. This can allow us to develop hypotheses related to the ecological and biogeochemical roles played by this group. For this motility guild, type II secretion and the Glyoxylate shunt co-occur with both chemotaxis and flagellum with a high degree of specificity (average outgroup confidence of 0.35).

### 4.2. MAG vs. SAG guild comparison

We ran both our MAG and SAG datasets through our method to investigate the differences in guilds generated by these two different datasets. These datasets not only used different methodologies but also sampled different oceanographic regions. The MAG dataset was comprised of globally distributed samples, most notably 68 sampling sites from *Tara* Oceans (Sunagawa et al., 2015) spanning all major oceanographic regions (except the Arctic Ocean) and three depths from the surface (5 m) to the mesopelagic zone (600 m). The SAG dataset on the other hand was obtained from samples primarily located in the North Atlantic and Pacific Oceans at a mean depth of 70.7 m and was prefiltered (Pachiadaki et al., 2019). Thus, the expectation is that these different datasets will yield different guilds because they sampled fundamentally different communities. Indeed, while guilds related to DMSP, the C-P lyase pathway, motility, and rhodopsins (Supplementary Table 5) were identified in the MAG dataset, the SAG dataset generated guilds primarily related to the uptake of substrates (Supplementary Table 6).

A guild associated with the acquisition of phosphorus was identified in both datasets. In the SAG dataset, this guild comprised of four functions and 163 mapback genomes, which consisted of the C-P lyase complex (PhnGHIJ), CP-lyase operon (PhnFKLMNOP), CP-lyase cleavage (PhnJ), and a phosphonate transporter (PhnCED). The C-P lyase pathway has been shown to break down a variety of phosphonate bonds, including phosphonates associated with semi-labile high molecular weight dissolved organic matter (Metcalf and Wanner, 1993; White and Metcalf, 2004; Sosa et al., 2017). It is unsurprising to see the CP-lyases grouped together since they are co-located in a single operon. However, this guild served as another example that our method can extract well-known functional co-occurrences (our method does not take into account the co-location of genes within the genome). These four functions associated with the SAG phosphorus guild were also found together in one of the MAG guilds with 62 mapback genomes.

The guilds identified by our method were an emergent property of the dataset itself. This means that the absence of a known or potential guild in the model output does not necessarily mean that guild was not present in the dataset. Using a different collection of annotated genomes could potentially change the abundances of the functions within the dataset, which could greatly impact whether the method identified a specific group of functions as a guild or not. For example, we demonstrated that guilds with abundances of 2% or lower were difficult to consistently observe. Furthermore, as discussed above, *K* is a crucial free parameter that needs to be selected for each novel dataset to which this method is applied. We recommend constraining *K* using a similar heuristic approach to the one we described above or using other previously suggested methods such as the Akaike information criterion (deLeeuw, 1992; Bingham et al., 2009).

## 5. Conclusion

The co-occurrence of metabolic functions has long been studied in the field of biochemistry where metabolic pathways are elucidated. However, these studies are typically very labor-intensive and require cultured representatives. This can present an issue since only a small fraction of marine microbes have been cultured (Rappé and Giovannoni, 2003; Steen et al., 2019). Our method described in this study presents a way to generate hypotheses about co-occurring functions across large collections of genomes without relying on cultured representatives. These hypotheses might aid in future biochemical studies by providing targeted functions to test.

In addition to generating testable hypotheses, this method presents several potential future applications. One possibility is in assisting with genome annotation through the incorporation of hypothetical gene products that have not yet been functionally characterized. One recent study (Faure et al., 2021) developed a large-scale sequence similarity network to identify protein functional clusters (PFCs) and demonstrated the potential for characterizing PFCs of previously unannotated proteins and correlating them with multiple environmental variables. Rather than focusing on whole community functional composition, our method identifies collections of ecologically relevant functions that are found to co-occur within assembled and isolate genomes. Using our method, one could construct a dataset composed of a mix of annotated and unannotated genes/proteins. Any mapback genomes identified for those hypothetical functions would be excellent culture candidates for characterizing that hypothetical gene. This method offers the potential to significantly refine the targeting of these culturing efforts to make them more nimble and more cost-effective.

Understanding microbial metabolic functional guilds is an essential step in describing microbial communities based on their metabolic activity, particularly for key heterotrophic communities. Rather than focusing on the functional composition of the entire community, our method identifies collections of co-occurring functions that form the building blocks of a community's functional structure. Defining the community as such will allow us to develop improved numerical ecosystem models that capture these metabolic capabilities. In addition, it will help us to better build and validate models, such as the trait-based ecosystem model GENOME described in Coles et al. (2017) study, that directly simulated the metagenomes and metatranscriptomes of communities. Furthermore, because our approach is phylogenetically independent, it also provides the ability to disentangle analyses of function and phylogeny when assessing the structure of a given community. This provides a window into the level of functional redundancy present both within a single guild and across the community as a whole. Additionally, our approach generates hypotheses about potential co-occurring metabolic functions that can be tested experimentally. Furthermore, since we demonstrate that this approach works for both MAG and SAG genomes, this method offers the ability to characterize the genomic potential of uncultured organisms from a wide range of studies.

## Data availability statement

Publicly available datasets were analyzed in this study. This data can be found here: https://github.com/LevineLab/AB-guilds_model; https://www.ncbi.nlm.nih.gov/bioproject/?term=PRJNA391943 BioProject ID PRJNA391943, https://mmp.sfb.uit.no/databases/; https://www.ncbi.nlm.nih.gov/bioproject/572885 BioProject ID PRJEB33281.

## Author contributions

RR and NL designed the project. RR, SH, NL, and JB developed and tested the AB model. RR and BT compiled the datasets and conducted the taxonomic classification, quality assessment, and phylogenetic analysis of the genomes. RR conducted the model simulations and guild analyses. All authors contributed to the writing of the manuscript, article, and approved the submitted version.
