# Supplementary material for: Identification of microbial metabolic functional guilds from large genomic datasets
Source: Front Microbiol. 2023 Jun 30;14:1197329. doi: 10.3389/fmicb.2023.1197329 (PMC10348482; doi:10.3389/fmicb.2023.1197329)
Supplement: Supplementary file 1 [file Data_Sheet_1.pdf]

1 Extended Methods

1.1 Phylogeny of Datasets

We constructed phylogenomic trees for both the composite and SAG only datasets as well as for the guilds presented in main text Table 2. Supplemental Figures 1-4 show the trees, annotated at the highest taxonomic classification with 2 or more distinct phylogenetic groups. We observed that the composite dataset that included globally sourced MAGs, isolates, and SAGs was much more diverse with 51 distinct bacterial phyla than the SAG only dataset which represented just 9 bacterial phyla (see Supplemental Figures 1&2). We also found that the phylogenetic diversity of our guilds varied. For example, the DMSP guild we identified was highly conserved within the Alphaproteobacteria, spanning just 4 distinct bacterial families with the majority of genomes coming from the family Rhodobacteraceae. On the other hand, the motility guild identified was diversely represented across 9 bacterial orders with the order Enterobacterales comprising the bulk of the associated mapback genomes.

We also computed ANI and AAI values for the composite dataset to numerically assess its diversity. We found that only 0.52% of the possible genome pairs produced a non-NaN ANI value, suggesting that this dataset was not appropriate for ANI based analysis. Instead, we used AAI, which is more suitable for genomes that are more phylogenetically distant from one another. After excluding values outside of FastAAI's defined range of 30-90%, we found that on average, a given genome pair had an AAI value of 39.1%. The full distribution of AAI values is shown in Supplemental Figure 5. We also assessed the AAI values between pairs of mapback genomes for each of our 10 guilds. To test this against the full dataset, we developed a Monte Carlo style simulation to approximate the distribution of average AAI values for a comparable subset of random genomes from the composite dataset. To accomplish this, we took 1,000 random subsets of 100 genomes – similar to the average number of genomes in a 5 function guild – and computed the average AAI value of all possible genome pairs. The resulting distribution can be seen in Supplemental Figure 6 with the individual guild average AAI values overlaid as vertical lines. We see that our guilds span the distribution with some guilds that have higher AAI values than average (more phylogenetically conserved) and some that have lower AAI values than average (less phylogenetically conserved). This suggests that our method is finding guilds which are sometimes phylogenetically conserved and sometimes not. As confirmation that it was possible to find 100 genomes with higher AAI values (more similar set of genomes), we took the 100 genomes with the most non-NA ANI values and computed their average AAI value. For this group, we found a much higher average value of 50.7% compared to the 39.1% value of the overall dataset.

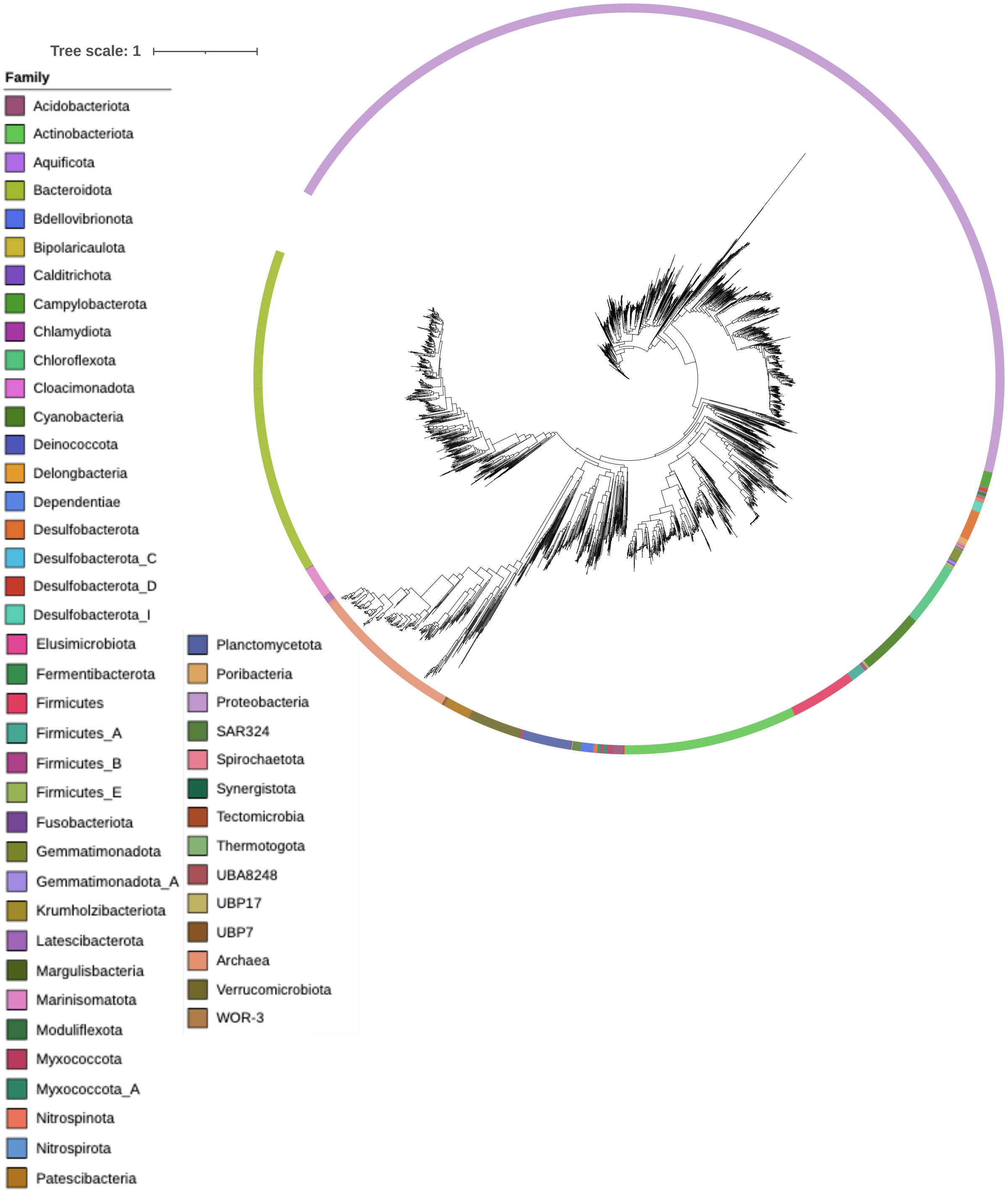

Supplemental Figure 1: *Phylogenomic tree of the full composite dataset consisting of 3,840 genomes including MAGs, SAGs, and isolate genomes. This tree presents 3,775 of those genomes (see Results) that represent 51 unique bacterial phyla and 2 archaeal phyla. For clarity, the two archaeal phyla have been collapsed simply into an "Archaea" designation.*

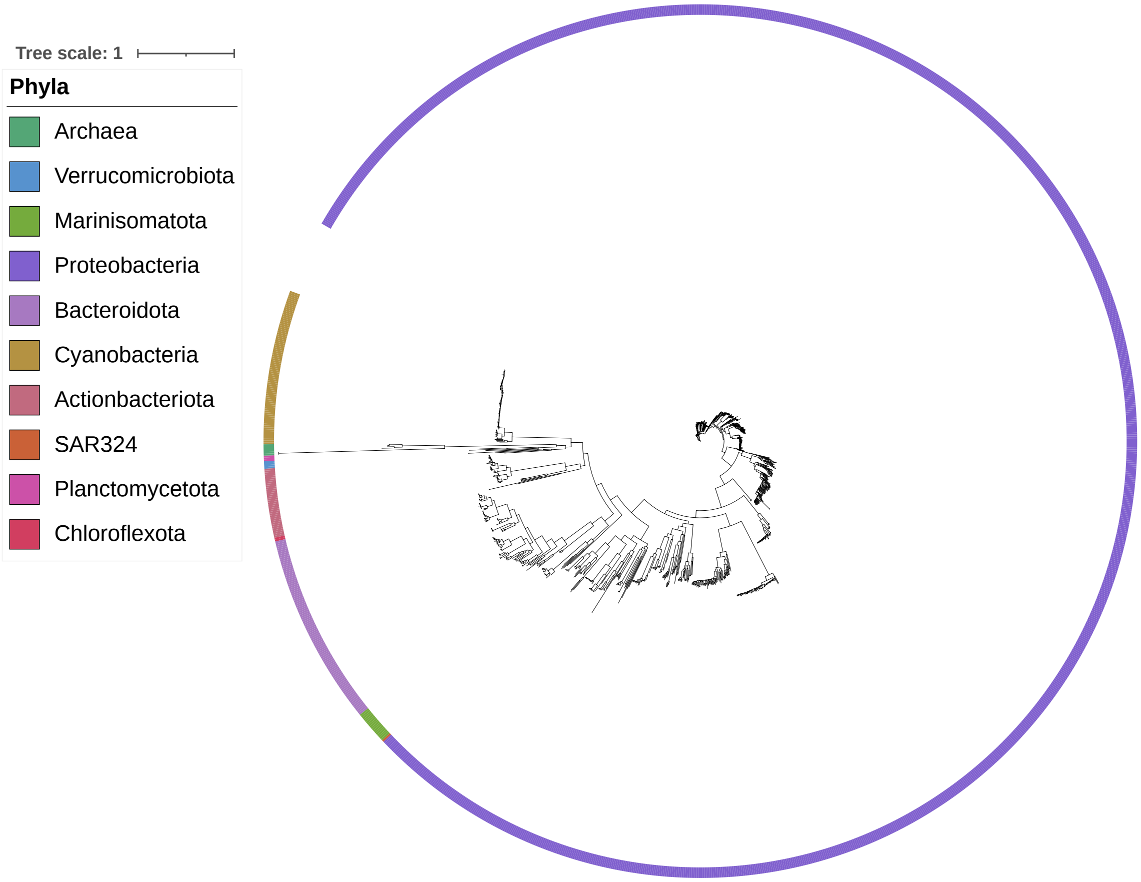

Supplemental Figure 2: *Phylogenomic tree for the SAG genomes sources and quality filtered from the GORG-Tropics expedition constituting 1,733 genomes. This tree presents 1,415 of those genomes (see Results) that represent 9 unique bacterial phyla as well as 2 archaeal phyla that are collapsed simply to the designation "Archaea".*

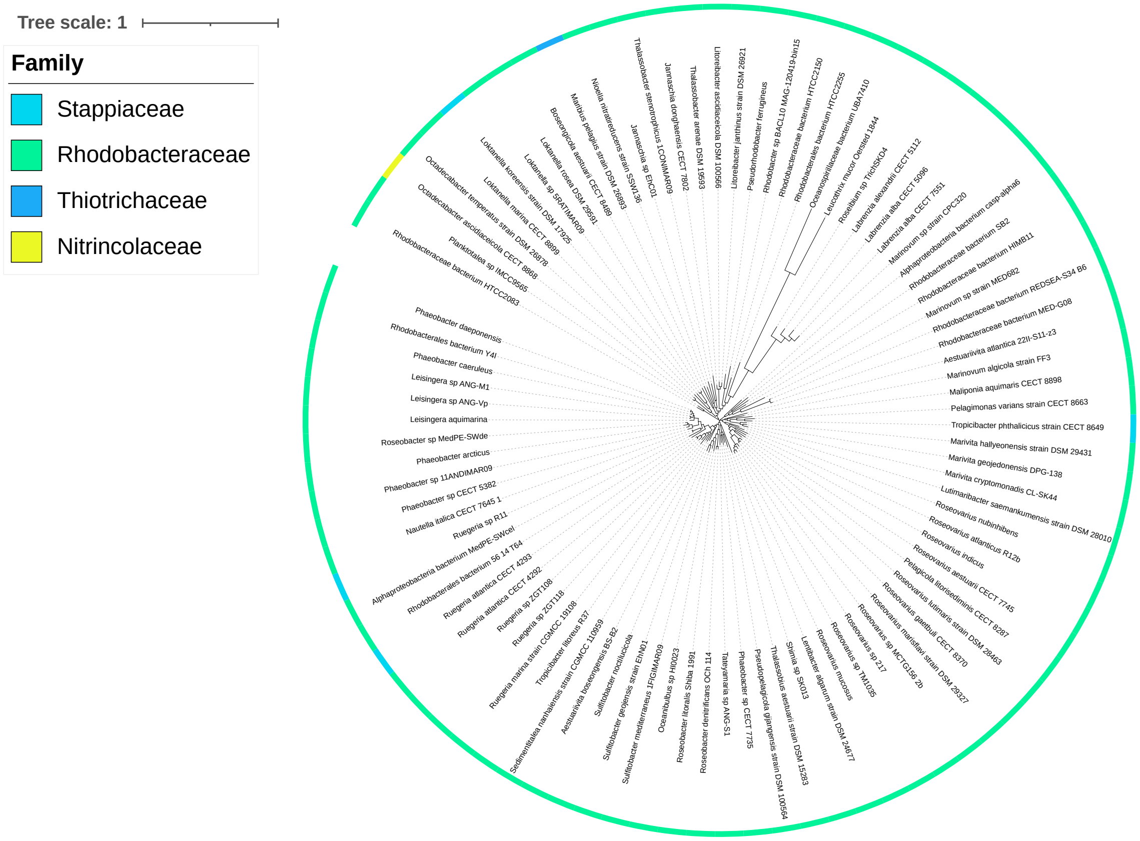

Supplemental Figure 3: *Phylogenomic tree for the DMSP guild as defined in the main text (see bolded functions in main text Table 2). This guild is distributed across 4 bacterial families, primarily Rhodobacteraceae.*

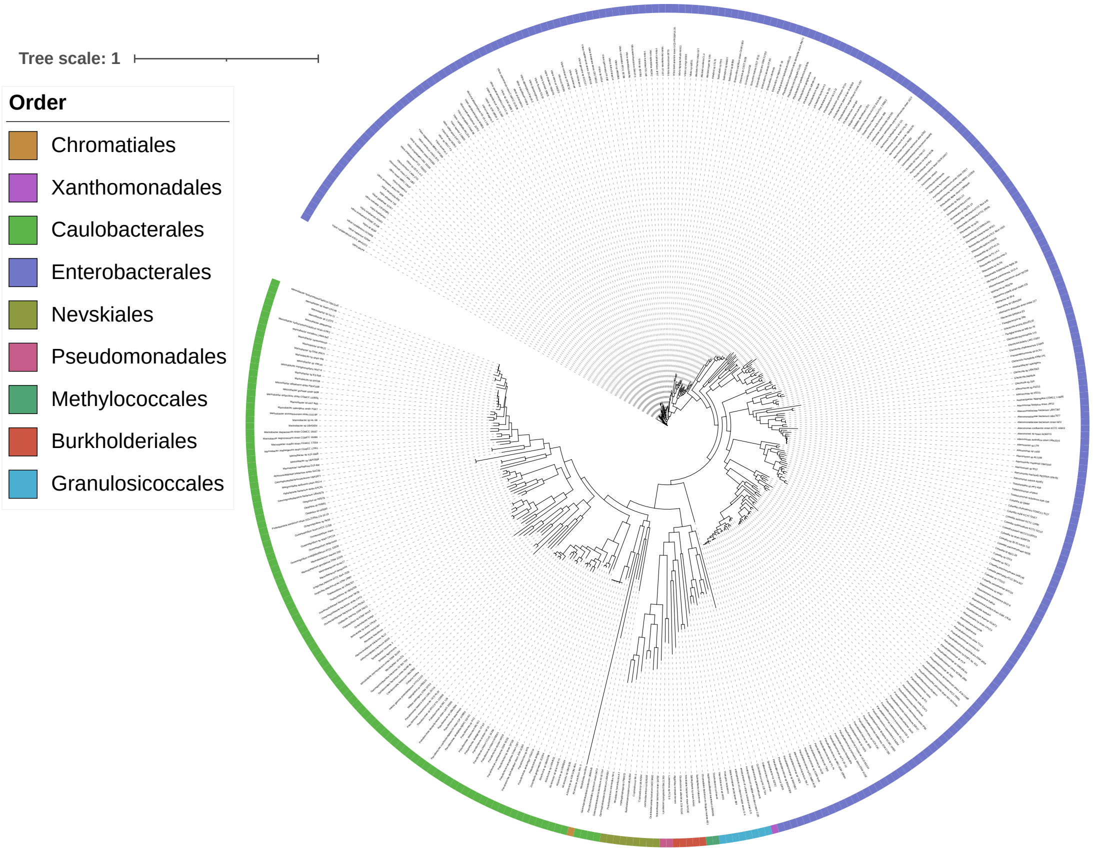

Supplemental Figure 4: *Phylogenomic tree for the motility guild as defined in the main text (see bolded functions in main text Table 2). This guild is distributed across 9 bacterial orders, most notably in the Enterobacterales and Caulobacterales.*

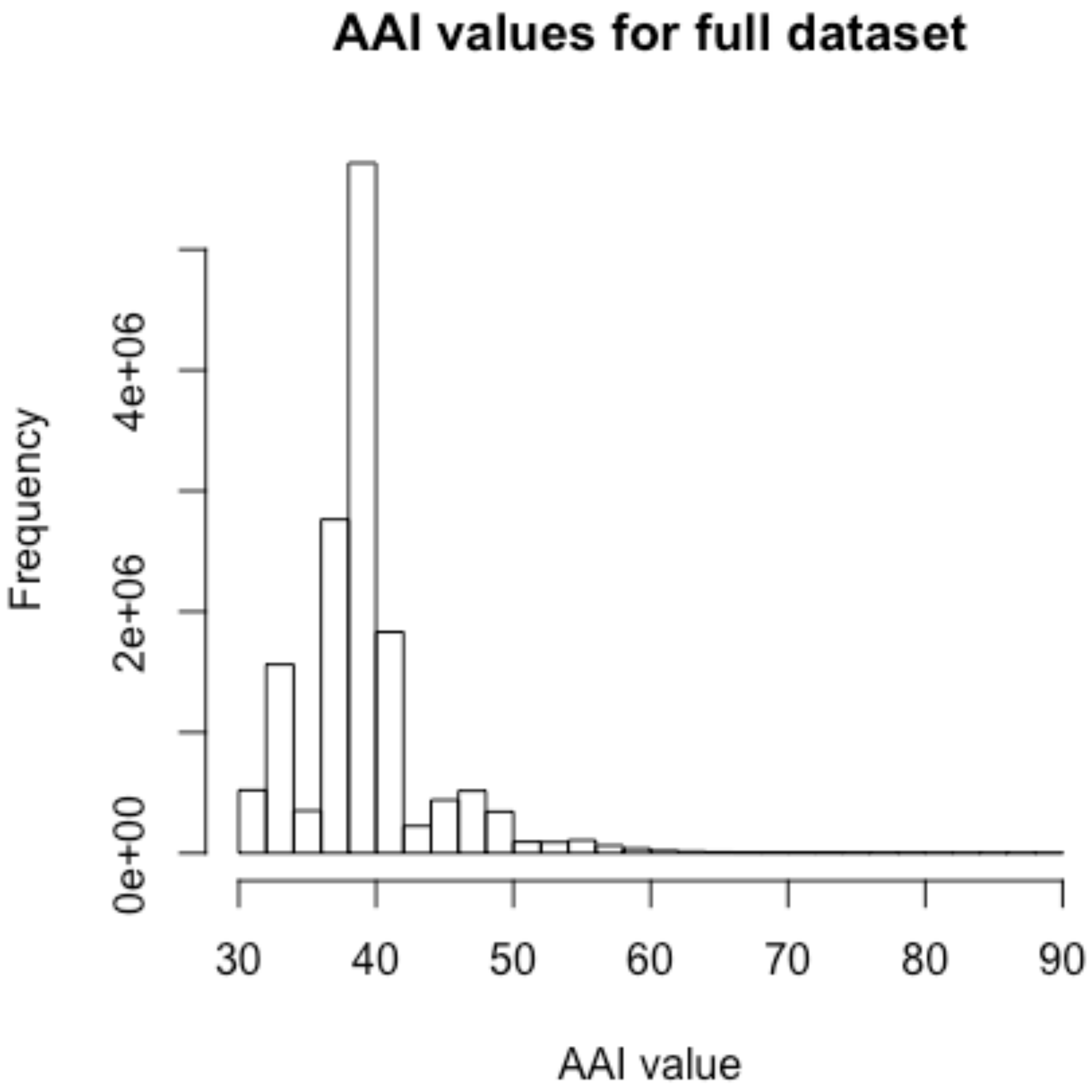

Supplemental Figure 5: *Total distribution of AAI values between 30% and 90% for all genome pairs in our composite dataset of 3,840 genomes. On average, a given genome pair had an AAI value of 39.1%.*

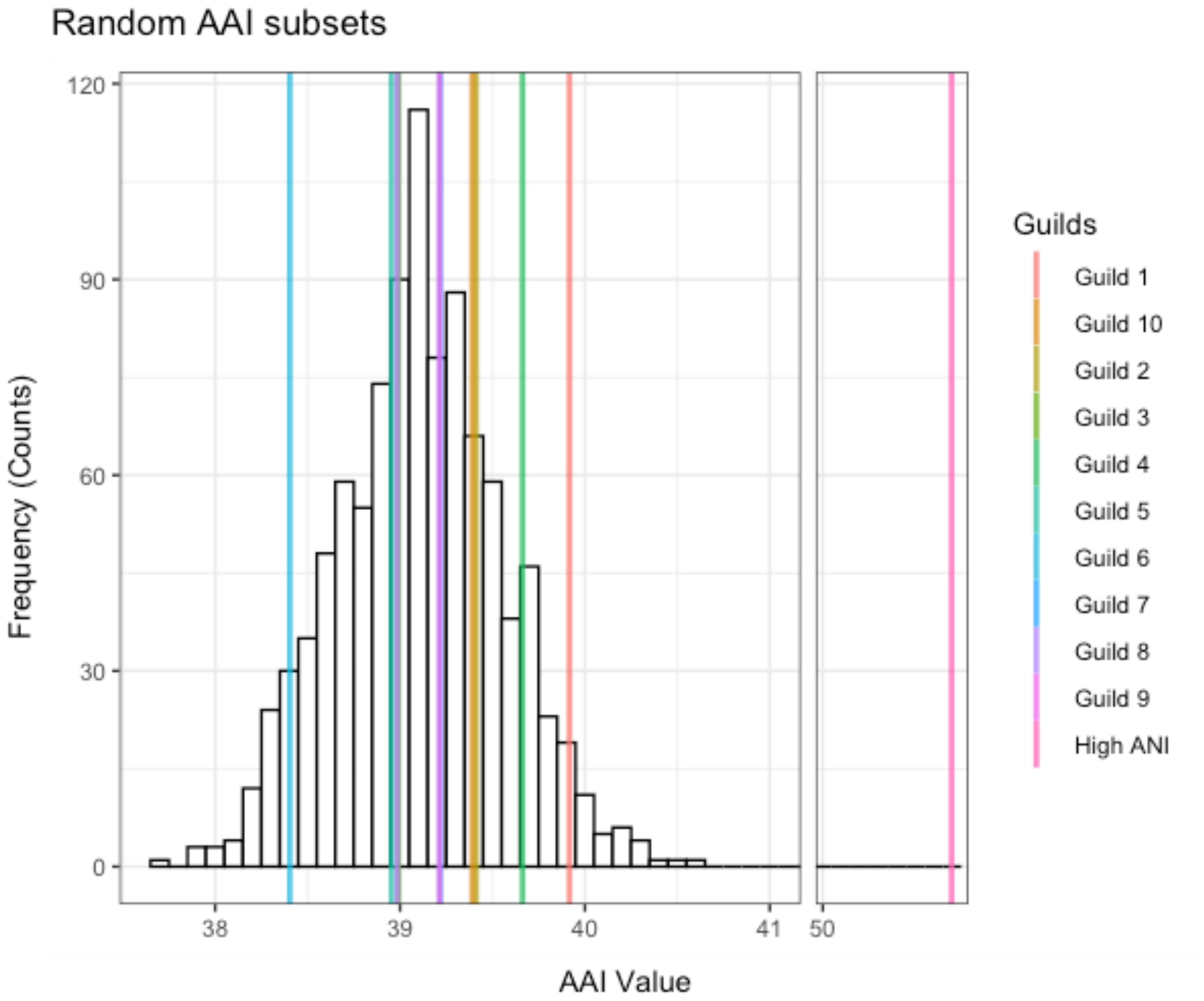

Supplemental Figure 6: *Histogram of average AAI values for our Monte Carlo style simulation of 1,000 sets of 100 random genomes. The AAI values for all genome pairs in each random set were averaged to construct the distribution in white bars. In addition, we computed the average AAI value for all pairs of genomes in each of our 10 guilds and overlaid those with colored vertical lines. The High ANI line that is shown to the right of the plot break shows the AAI value for the 100 genomes with the most non-NA ANI values (i.e., the most similar set of 100 genomes). The axis break was produced using ggbreak (Xu et al., 2021).*

1.2 Extended AB Method

Below we present the complete procedure for selecting functional guilds from an Aspect Bernoulli model:

1. Calculate Aspect Bernoulli estimates $\hat{\Gamma}$ and $\hat{\beta}$, main text Eq. 1
2. Calculate $r_{fk} = \frac{1}{G}\sum_{g=1}^{G}\frac{\Gamma_{gk}\beta_{kf}}{V_{gf}}$, main text Eq. 2 and Eq. 3
3. For each aspect $k = 1, \cdots, K$:

a. Calculate $A_k \subseteq \{1,\cdots,G\}$ as the set of genomes such that the following is true:
   - For each $k' \in K,\ k' \neq k$, $\Gamma_{g,k} > \Gamma_{g,k'}$
   - $\Gamma_{g,k} > \frac{2}{K}$
b. Compute $q_{fk} = \frac{\sum_{g \in A_k} Y_{gf}}{\frac{1}{F}\sum_{f=1}^{F}\sum_{g \in A_k} Y_{gf}}$, main text Eq. 4
c. Form the score $s_{fk} = q_{fk} \cdot r_{fk}$, main text Eq. 5
d. Denote as $\{o_{ik}\}_{i=1,\cdots,F}$ the decreasing order of $\{s_{fk}\}_{f=1,\cdots,F}$ (such that $s_{o_{\cdot,k},k}$ is sorted from high to low). Next, denote as $\tilde{Y}_{j,\cdot} = Y_{j,o_{f,\cdot}}$ the rearranged version of the row $Y_{j,\cdot}$ and proceed to Option 1 or 2.
   - **Option 1: Fixed guild size** Calculate B_k to be the set of row indices j such that $\tilde{Y}_{j,1:5}$ are all equal to 1.
   - **Option 2: Data guild size**
     - For each $m = 1,2,\ldots,F$ calculate w_m to be the set of row indices j such that $\tilde{Y}_{j,1:m}$ are all equal to 1. Set $\tilde{m}$ to be the largest value of m such that $|w_m| \geq 100$.
     - Set B_k to be equal to $w_{\tilde{m}}$ and set functional guild F_k to be equal to $o_{1:\tilde{m},k}$.

The underlying assumptions of AB are twofold. First, entry $Y_{g,f} \in \{0,1\}$ (the presence or absence of a function in a genome in our dataset) is a random Bernoulli realization of an underlying scalar probability $V_{g,f} \in [0,1]$. Second, the matrix of probabilities $\{V_{gf}\}_{g=1,\ldots,G,f=1,\ldots,F}$ has an underlying low-dimensional representation, and each entry is assumed to be a convex combination of K probabilities:

$$V_{gf} = \Gamma_{g\cdot}\beta_{\cdot f}\ , \qquad \textit{Eq. 1}$$

where β and Γ are two matrices which relate to a latent variable Z_{gfk} – where g denotes genome, f denotes function, and k denotes aspect (see Terminology box for definition). The latent variable Z_{gfk} encodes whether aspect k is the active aspect for genome-function pair gf (that is, only one 1 for each gf pair; zero otherwise). With this latent variable, we can describe the matrices β and Γ more precisely:

- $\Gamma_{gk} = P(Z_{gfk} = 1)$ quantifies how strong the kth aspect is, within each genome g.
- $\beta_{kf} = P(Y_{gf} = 1 | Z_{gfk} = 1)$ is the probability that function f is present given that the kth latent aspect is present for a given genome-function pair.

A visual schematic of the Aspect Bernoulli model can be seen in Supplemental Figure 7.

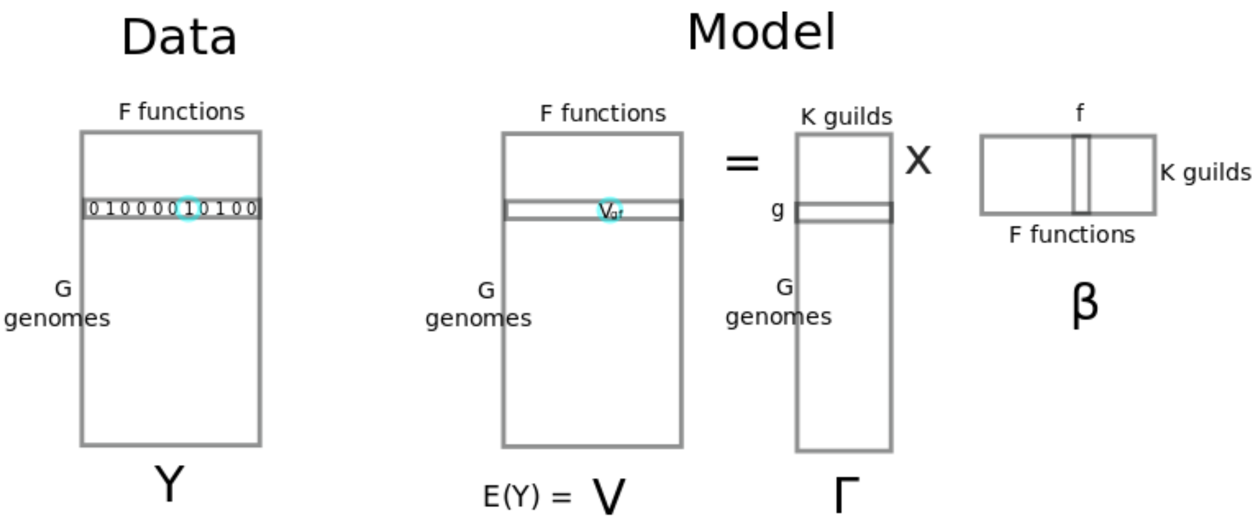

Supplemental Figure 7: *Visual schematic of the model procedure that shows how we model our data matrix Y as a matrix V of Bernoulli probabilities that we then decomposed into the two matrices Γ and β to create a low-dimensional representation of Y.*

1.3 Extended Classical Methods

We ran the hierarchical clustering method *clustergram* at a cut height of 1 (Supplemental Figure 8) in addition to the cut height of 0.9 (main text Figure 3). The choice of linkage value determines how sensitive the merging step is when determining whether to adjoin nodes together in a cluster. So, increasing the linkage from 0.9 to 1 had the primary effect of enlarging the cluster sizes from 5.8 to 11.1 functions per guild while reducing the total number of clusters from 30 to 17.

We also tested the use of a dynamic cut height based on the topology of the tree using the dynamicTreeCut package v1.63.1 on a complete linkage dendrogram generated by hclust and analyzed with the dendextend v1.17.1 package (Galili, 2015) with R v4.2.3. With this dynamic cut height we identified 19 distinct guilds averaging 8.8 functions (range 5-19) with an average of 47.6 mapback genomes per guild (range 0-391). However, 26.3% of the guilds (N=5) still hada no mapback genomes. Though this is overall a marked improvement over the static height clustering values, it is still substantially less than those of the AB guilds (see main text Section 3.4).

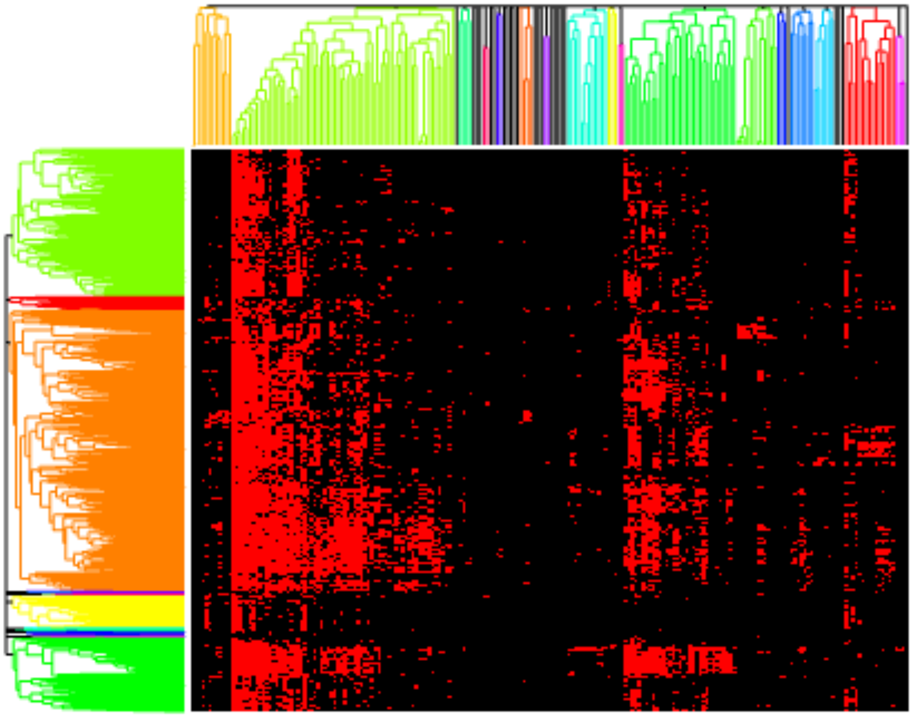

Supplemental Figure 8: *Resulting dendrogram from running clustergram on our composite dataset with a cut height of 1 (red = present, black = absent). The rows (genomes) and columns (functions) were both clustered using the Jaccard distance metric.*

2 Extended validation of AB

2.1 Extended Simulated Data Analysis

To test the sensitivity of the Aspect Bernoulli (AB) model to the number of factors, K, we generated simulated datasets in which either one or three artificial guilds were added to the composite dataset (see Methods). For each artificial guild type, we created 100 datasets with individually inserted guilds drawn from nine combinations of guild size (i.e., number of functions) and abundance (i.e., number of genomes). The AB model was then run using K values ranging from 5 to 20. The results from the sensitivity tests are presented in Supplemental Table 2 for three values of K across our tested range ($K = 5,10,20$). Within each super column of Supplemental Table 2 (e.g., $K = 5$) three metrics (sub columns) are shown: Hit Rate, Extra Hits, and Multi Hits (which we define below). The full set of values for each of these three metrics is plotted from $K = 5$ to $K = 20$ for simulations with three and one artificial guilds respectively for four of the size/abundance combinations in Supplemental Figures 9&10. Below, we present the results from the three artificial guild simulated data.

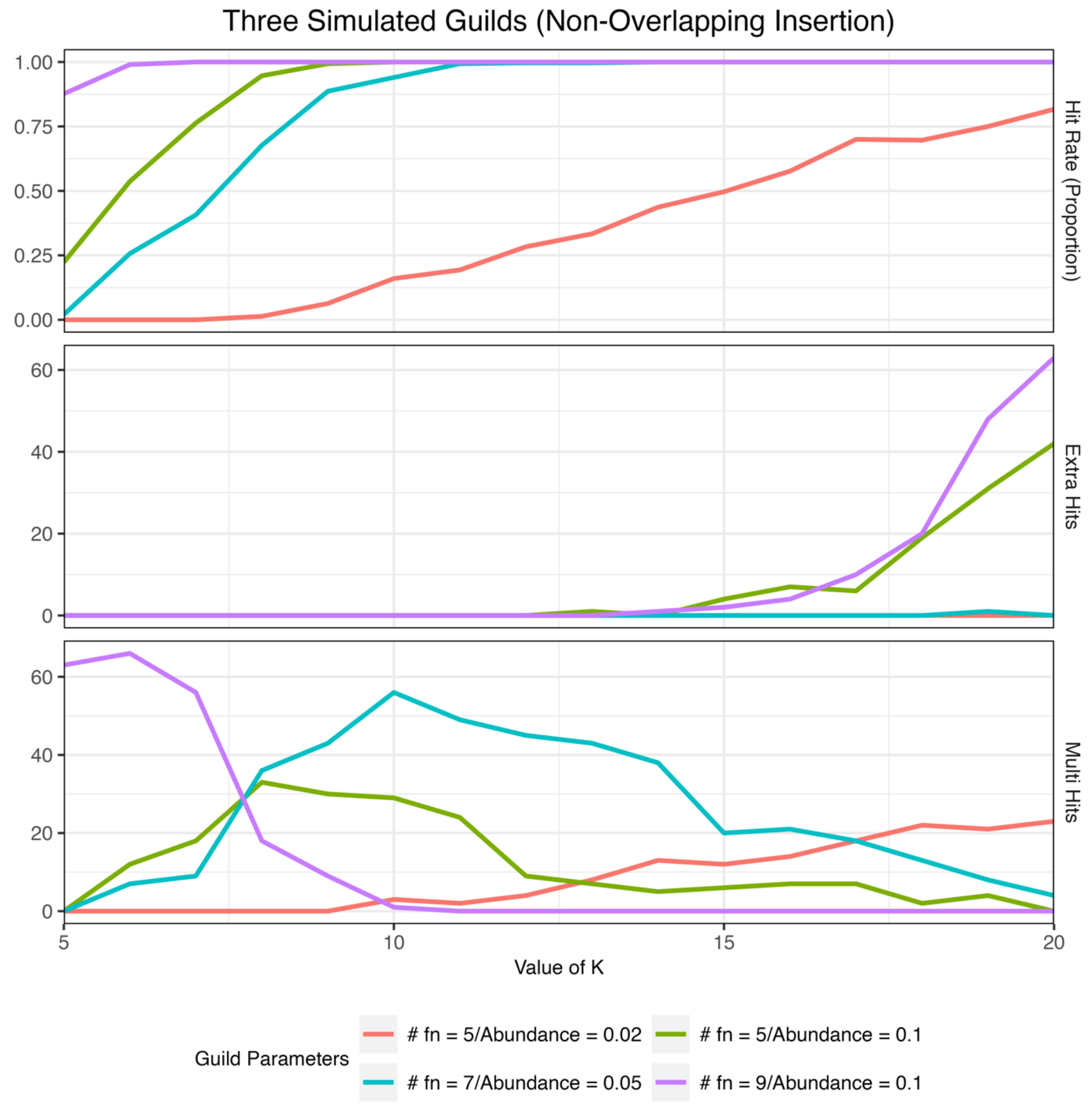

Supplemental Figure 9: *Simulated data metric values for 100 simulations with three artificial guilds across the tested range of K's (K=5-20): Hit Rate as a proportion (top panel), Extra Hits (middle panel), and Multi Hits (bottom panel).*

As described in the main text, Hit Rate describes the overall frequency of an artificial guild being identified by the method (i.e., appearing in the top 15 functions of an aspect). One can think of this as the recovery rate of an artificial guild. For example, at $K = 5$ we see that an artificial guild of size 5 with 2% abundance was never found. As shown in Supplemental Figure 9, the hit rate for this guild improved as we increased K, reaching 16.0% at $K = 10$ and 81.7% at $K = 20$ (Supplemental Table 2), substantially improving our ability to recover this rare guild. Similarly, as the number of functions contained within the 2% abundant artificial guild increased from 5 to

9, the recovery rate increased as well (aside from the case of $K = 5$ where it remains undetectable) (Supplemental Table 2). This trend held across all three abundance levels.

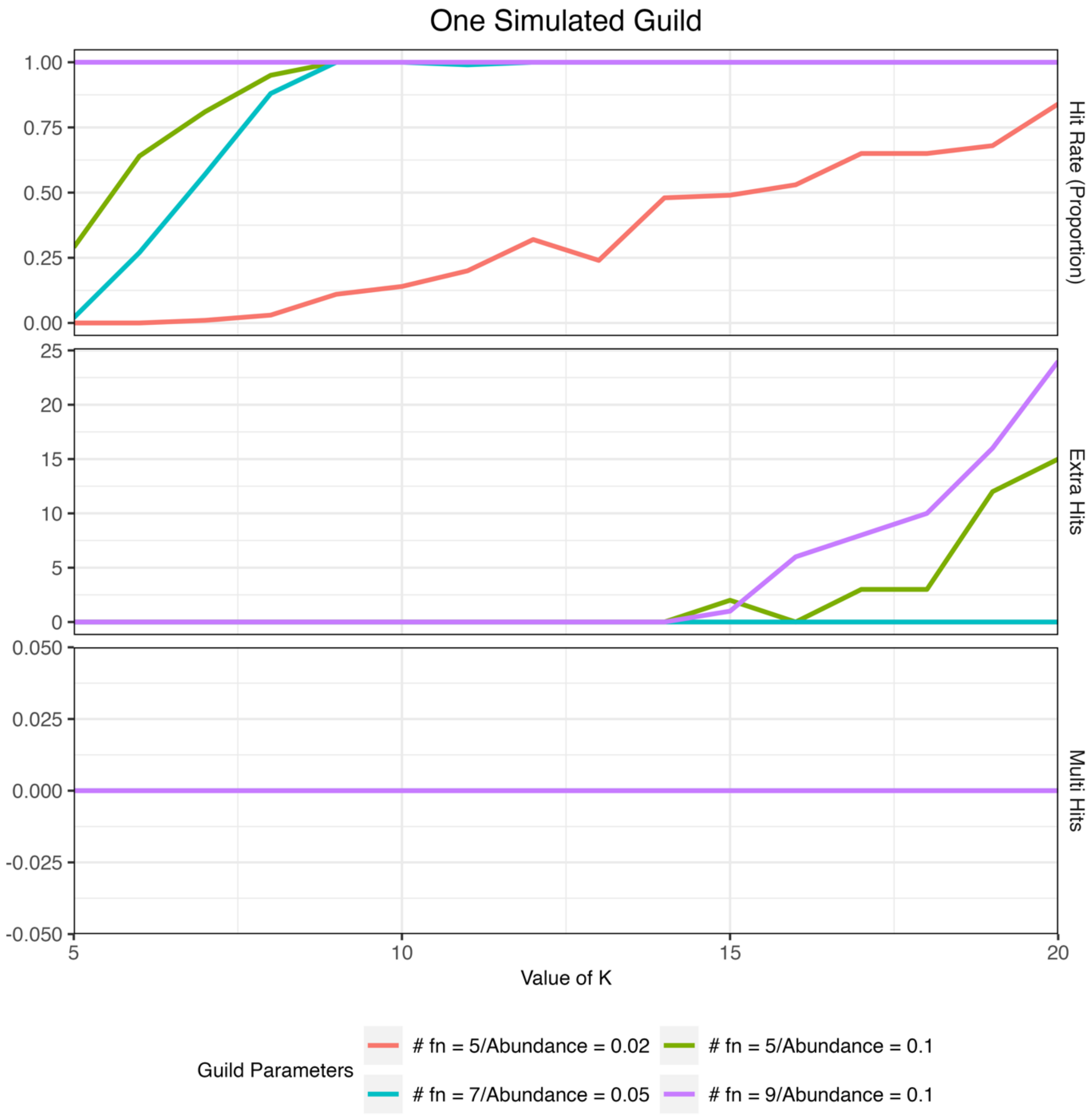

Supplemental Figure 10: *Simulated data metric values for 100 simulations with a single artificial guild across the tested range of K's (K=5-20): Hit Rate as a proportion (top panel), Extra Hits (middle panel), and Multi Hits (bottom panel). As seen in the bottom panel, there are no Multi Hits for the single guild simulations because you must have 2+ guilds to register one as defined (Supplemental Section 2).*

For each run, we also calculated Extra Hits (where an artificial guild appears in at the top of the function list in multiple aspects) and Multi Hit (where two or more artificial guilds occur together at the top of the function list in a single aspect). The presence of Extra Hits in these datasets is

interpreted as overfitting by the Aspect Bernoulli algorithm since each artificial guild should appear only once and be completely intact. At $K = 5$ and $K = 10$, zero Extra Hits were observed for any of our nine size/abundance combinations. However, at $K = 20$, 4.7% of the total observed hits across all guild parameter combinations were Extra Hits, reaching as much as 17.4% of the total observed hits for guilds of size 9 and abundance of 10%. In other words, when K was high and the artificial guild was abundant, the method identified additional underlying structure in the composite dataset and combined this with that abundant guild.

The presence of Multi Hits is interpreted to be an underfitting by the Aspect Bernoulli algorithm since each guild should be in its own distinct factor. For large, high abundance guilds (e.g. size 9, 10% abundance guild in Supplemental Table 2) which have high Hit Rates even at low K values, we observed a large number of Multi Hits when K was small (evidence for underfitting), with 21.0% of the total observed hits being Multi Hits when $K = 5$. Multi Hits then decreased as K increased as a result of more accurate fitting as the model was able to separate the artificial guilds into individual factors. For artificial guilds with medium size and abundance, the Hit Rate was low at low K. When this was the case, Multi Hits were also initially low. For these artificial guilds, both Hit Rate and Multi Hits increased as K increased. Once the Hit Rate approached 100%, the Multi Hits began to decline back to zero with further increases in K (Supplemental Figure 10). For example, in the case of a guild with 7 functions and a 5% abundance, the Multi Hits increased from none at $K = 5$ to 18.7% at $K = 10$ before decreasing again to just 1.3% of hits at $K = 20$ (Supplemental Table 2). At the same time, the Hit Rate increased from 2.0% at $K = 5$ to 94.0% at $K = 10$ and 100% at $K = 20$. Low size and abundance guilds showed a similar pattern to medium size guilds but the transitions were shifted to higher values of K.

Finally, we checked whether our guild hits in each run were unique, or if some of the hits were duplicate occurrences of the same artificial guild. For each simulated dataset that produces Extra Hits, all of the unique guilds always appeared, as opposed to multiple duplications of the same artificial guild.

2.2 Randomly Inserted Artificial Datasets

The results described above and presented in Supplemental Table 2 used non-overlapping guilds such that each genome could only be a member of one artificial guild. We also generated a second dataset type in which the artificial guilds were inserted randomly rather than in a strictly non-overlapping fashion, such that any given genome could be a member of any number of artificial guilds (Supplemental Table 3). These overlapping datasets were generated using three simulated guilds and the same nine size/abundance combinations as shown in Supplemental Table 2. Overall, we found that the Hit Rates and Extra Hits for the overlapping guilds were remarkably similar to the values from the non-overlapping artificial guilds, but the frequency of Multi Hits increased on average by 116.1% at $K = 5$, 83.1% at $K = 10$, and 79.7% at $K = 20$. We conclude this is most likely due to the random insertion of guilds, which can create a stronger linkage between artificial guilds than if only one guild is allowed per genome. As discussed in Supplemental Section 2.1, for guilds with medium size and abundance, the increase in Multi Hits at low K values corresponded to increasing Hit Rates. Once the Hit Rates neared 100%, the Multi Hits decreased to zero as the model began to distinguish those guilds. The full range of values are presented for four of the

size/abundance combinations for 100 simulated datasets with three randomly inserted guilds in Supplemental Figure 11.

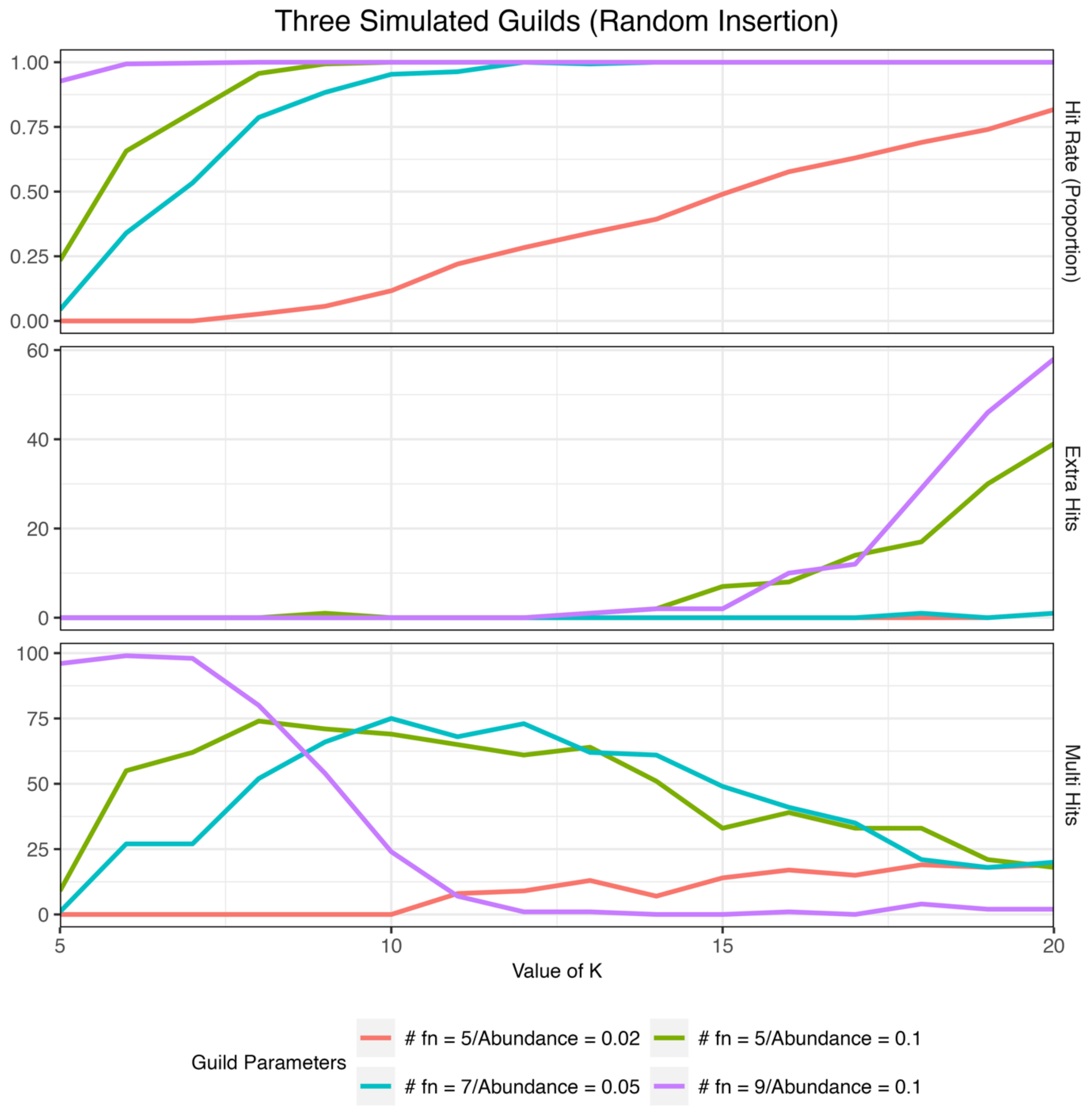

Supplemental Figure 11: *Simulated data metric values for 100 simulations with three artificial guilds randomly inserted into the dataset across the tested range of K's (K=5-20): Hit Rate as a proportion (top panel), Extra Hits (middle panel), and Multi Hits (bottom panel).*

2.3 Extended Analysis of the Impact of K

In addition to impacting the frequency with which we identify guilds of different sizes and abundances, the choice of K also influences the number of mapback genomes associated with each guild. As mentioned in the Methods section, for each aspect k, the pipeline provides a score for

every function in the dataset. The user must then make a decision as to the subset of functions to include within each functional guild. We investigated several different choices for determining guild functions (*see Methods*). When K was low (e.g., < 10), we observed higher numbers of mapbacks on average (Supplemental Table 7). We attribute this to the fact that, at low K, the pipeline preferentially identified guilds of functions that were more abundant in the dataset. Specifically, guilds identified at $K = 5$ averaged 362 mapback genomes (range 61-1,295) compared to 116 genomes (range 11-468) for guilds identified at $K = 10$ and 54 genomes (range 0-210) for guilds identified at $K = 20$. This analysis was conducted by defining the guilds as the top 5 functions in each aspect.

The number of functions included within each guild has a large impact on the number of mapback genomes, as expected (Figure 3). For all aspects, as the number of functions in a guild decreased, the number of mapback genomes increased. In the most extreme case, when a guild was defined by just the top two functions, guilds identified at $K = 10$ in the composite dataset averaged 301 mapback genomes (range 79-694). For many guilds, we observed a plateauing of mapback genomes across a range of guild sizes indicating the presence of a robust guild in which there was a strong co-occurrence of functions (e.g., if function A then also functions B and C). Specifically, decreasing the number of functions required to be a member of the guild did not substantially change the number of mapback genomes.

We also compared the number of mapback genomes we found among our probabilistic representatives (defined as the top 5 scoring functions in each aspect) to the number of mapback genomes across the full dataset. In most instances, there were additional mapback genomes in the full dataset what were not included in the probabilistic representatives. For the composite dataset, when $K = 10$, there was on average a 33% increase (range 0-134%) in the number of mapback genomes found within the entire dataset as compared to just within the probabilistic representatives. This occurred because genomes can be members of multiple guilds. For the probabilistic representative identification, each genome is only assigned to the single aspect with which it is most strongly associated, according to that genome's Γ vector.

3 Speed and Stability

3.1 Computational speed

In this section, we briefly examine the computational speed of the EM algorithm (Bingham, Kabán, and Fortelius 2009) by running the algorithm once on our main dataset and examining the objective value o_i across algorithm iterations $i = 1,2,\cdots$ (Supplemental Figure 12). In our analysis (see Results), we stopped the algorithm at a fixed number of iterations (2,000), which took about 1,320 seconds on a single core of an M1 processor. More generally, we recommend the conventional stopping rule for this algorithm class – stopping when the relative improvement of the objective value $(o_i - o_{i-1})/o_{i-1}$ is sufficiently small, e.g., 10^{-6}.

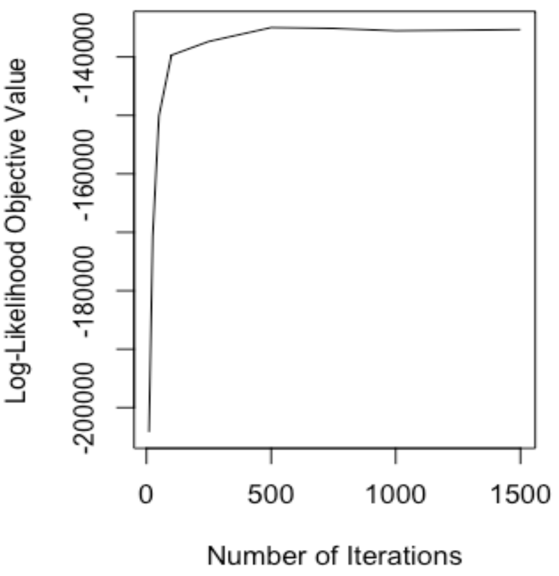

Supplemental Figure 12: *Value of the MLE estimator for runs of the AB that only vary in number of iterations used.*

3.2 Numerical Stability

In estimating the AB model, we attempt to maximize a non-convex objective. For a better chance at finding a global maximum, the EM algorithm must be run multiple times with new random initializations, and the final estimate can be taken from the run with the highest final objective value. Even using multiple restarts, obtaining a near-global optimum is still computationally difficult. In the following analysis, we further investigated how numerically stable the estimated guilds are, from two independent model estimates.

We produced each of the two model estimates as an approximate result of 30,000 random initializations. Specifically, we first ran the EM algorithm many times (30,000 random initializations) for a short amount of time (300 iterations) in order to identify an especially promising initialization, which we then reran through the EM algorithm until we reached numerical convergence.

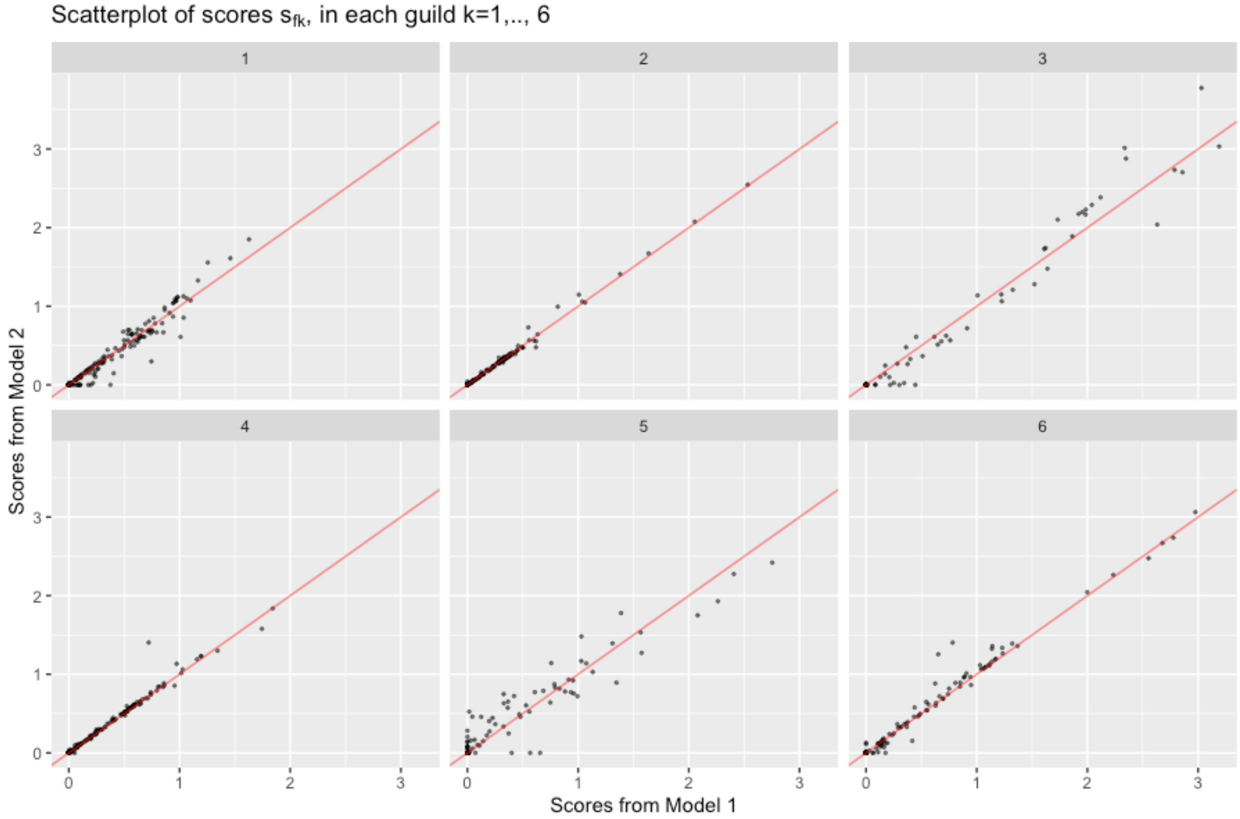

Supplemental Figure 13: *Comparison of scores for matched guilds from two independent AB model estimates using a two-step approach that identified good initialization states and then ran the EM algorithm for many steps for those states. We see that the scores lie along the 1:1 line (red), showing that the guilds are relatively stable across model estimates.*

Next, the six aspects from each of the two model estimates needed to be numerically matched using an appropriate permutation of one set of six aspects to closely resemble the other set. For this matching, we used the Hungarian algorithm (Kuhn, 1955) to find the best permutation of one model's estimated $\{\Gamma_{\cdot,k}\}_{k=1}^{K}$ to the other's. Matching using $\{\beta_{\cdot,k}\}_{k=1}^{K}$ produces the same result.
The final comparison of the two model estimates is presented in Supplemental Figure 13 and Supplemental Table 8. Supplemental Figure 13 shows six scatterplots (one for each aspect k) of the scores $\{s_{fk}\}_{f=1}^{F}$ from the two model estimates. The points in each scatterplot have a close but imperfect fit to the $y = x$ line. Next, Supplemental Table 8 shows the six functional guilds produced using our proposed pipeline (option 2 of Supplemental Section 1) from the two model estimates. Of note, Guilds 4 and 6 are perfectly conserved across models 1 and 2 and strongly resemble the DMSP and motility guilds, respectively, that we present in the main text (see Discussion). Though the matched scores do not fall precisely along the 1:1 line shown in red on

each plot, the guilds in Supplemental Table 8 were very consistent between models, and the scores still showed a strong linear trend.

Supplemental Table 1: *Complete function list used in this study, as listed in the KEGG decoder program used for function/pathway annotation* (Graham et al., 2018). *Functions are listed as they appear in KEGG decoder in a computer readable format (no spaces, font styles, or reserved characters).*

glycolysis	NAD-reducinghydrogenase	CP-lyasecomplex	riboseSBP
gluconeogenesis	NADP-reducinghydrogenase	CP-lyaseoperon	erythritolSBP
TCACycle	NiFehydrogenaseHyd-1	TypeISecretion	putativexylitolSBP
NAD(P)H-quinoneoxidoreductase	thiaminbiosynthesis	TypeIIISecretion	inositolSBP
NADH-quinoneoxidoreductase	riboflavinbiosynthesis	TypeIISecretion	inositol-phosphateSBP
F-typeATPase	cobalaminbiosynthesis	TypeIVSecretion	putativefructooligosaccharideSBP
V-typeATPase	transporter:vitaminB12	TypeVISecretion	glycerolSBP
Cytochromecoxidase	transporter:thiamin	Sec-SRP	putativesn-Glycerol3-phosphateSBP
Ubiquinol-cytochromecreductase	transporter:urea	TwinArginineTargeting	putativesorbitol/mannitolSBP
Cytochromeoubiquinoloxidase	transporter:phosphonate	TypeVabcSecretion	arabinosaccharideSBP
Cytochromeaa3-600menaquinoloxidase	transporter:phosphate	sulfateSBP	gamma-hexachlorocyclohexaneSBP
Cytochromecoxidase,cbb3-type	Flagellum	molybdateSBP	phospholipidSBP
Cytochromebdcomplex	Chemotaxis	molybdate/tungstateSBP	putativemultiplesugarSBP
RuBisCo	Methanogenesisviamethanol	tungstateSBP	putativesimplesugarSBP
CBBCycle	Methanogenesisviaacetate	nirate/nitriteSBP	lysine/arginine/ornithineSBP
rTCACycle	Methanogenesisviadimethylsulfide, methanethiol,methylpropanoate	bicarbonateSBP	histidineSBP

Wood-Ljungdahl	Methanogenesisviamethylamine	taurineSBP	glutamineSBP
3-HydroxypropionateBicycle	Methanogenesisviatrimethylamine	sulfonateSBP	arginineSBP
4-Hydroxybutyrate/3-hydroxypropionate	Methanogenesisviadimethylamine	phthalateSBP	glutamate/aspartateSBP
ammoniaoxidation	MethanogenesisviaCO2	ironiiiSBP	octopine/nopalineSBP
hydroxylamineoxidation	CoenzymeB/CoenzymeMregeneration	putrescineSBP	generall-aminoacidSBP
nitriteoxidation	CoenzymeMreductiontomethane	spermidine/putrescineSBP	glutamateSBP
dissimnitratereduction	Solublemethanemonooxygenase	putativespermidine/putrescineSBP	cystineSBP
DNRA	dimethylamine/trimethylaminedehydrogenase	mannopineSBP	l-cystineSBP
nitritereduction	PhotosystemII	2-aminoethylphosphonateSBP	arginine/ornithineSBP
nitricoxidereduction	PhotosystemI	glycinebetaine/prolineSBP	putativelysineSBP
nitrous-oxidereduction	Cytochromeb6/fcomplex	osmoprotectantSBP	branchedchainaminoacidSBP
nitrogenfixation	anoxygenictype-IIreactioncenter	hydroxymethylpyrimidineSBP	neutralaminoacidSBP
hydrazinedehydrogenase	anoxygenictype-Ireactioncenter	putativethaiminSBP	d-methionineSBP
hydrazinesynthase	Retinalbiosynthesis	maltose/maltodextrinSBP	putativeglutamineSBP
dissimilatorysulfate<>APS	Entner-DoudoroffPathway	arabinogalactanSBP	putativeaminoacidSBP
dissimilatorysulfite<>APS	Mixedacid:Lactate	raffinose/stachyose/melibioseSBP	putativepolaraminoacidSBP
dissimilatorysulfite<>sulfide	Mixedacid:Formate	alpha-glucosideSBP	oligopeptideSBP

thiosulfateoxidation	Mixedacid:FormatetoCO2&H2	glucose/arabinose SBP	dipeptideSBP
altthiosulfateoxidationtsdA	Mixedacid:Acetate	glucose/mannoseSBP	cationicpeptideSBP
altthiosulfateoxidationdoxAD	Mixedacid:Ethanol,AcetatetoAcetylaldehyde	trehalose/maltose SBP	nickelSBP
sulfurreductasesreABC	Mixedacid:Ethanol,Acetyl-CoAtoAcetylaldehyde(reversible)	trehaloseSBP	glutathioneSBP
thiosulfate/polysulfidereductase	Mixedacid:Ethanol,AcetylaldehydetoEthanol	n-acetylglucosamine SBP	peptides/nickelSBP
sulfhydrogenase	Mixedacid:PEPtoSuccinateviaOAA, malate&fumarate	cellobioseSBP	microcincSBP
sulfurdisproportionation	Naphthalenedegradationtosalicylate	n-diacetylchitobiose SBP	ironcomplexSBP
sulfurdioxygenase	BiofilmPGASynthesisprotein	putativechitobiose SBP	manganeseSBP
sulfitedehydrogenase	ColanicacidandBiofilmtranscriptionalregulator	l-arabinoseSBP	zincSBP
sulfitedehydrogenase(quinone)	BiofilmregulatorBssS	lactose/l-arabinoseSBP	iron/zinc/manganese/copperSBP
sulfideoxidation	ColanicacidandBiofilmproteinA	oligogalacturonide SBP	manganese/ironSBP
sulfurassimilation	Curlifimbriaebiosynthesis	alpha14digalacturonateSBP	manganese/zincSBP
DMSPdemethylation	Adhesion	putativealdouronateSBP	manganese/zinc/ironSBP
DMSdehydrogenase	Competence-relatedcorecomponents	methyl-galactosideSBP	cobalt/nickelSBP
DMSOreductase	Competence-relatedrelatedcomponents	d-xyloseSBP	biotinSBP
NiFehydrogenase	Competencefactors	xylobioseSBP	beta-carotene15,15-monooxygenase
ferredoxinhydrogenase	Glyoxylateshunt	multiplesugarSBP	rhodopsin

membrane-boundhydrogenase	Anapleroticgenes	d-alloseSBP	transporter:ammonia
hydrogen:quinoneoxidoreductase	Sulfolipidbiosynthesis	fructoseSBP	DMSPlyase(dddLQPDKW)

Supplemental Table 2: *Results of Aspect Bernoulli runs with three non-overlapping artificial guilds at three aspect numbers K=5,10,20. Hit Rate describes the percentage of hits observed out of all possible hits. Extra Hits represent runs where an artificial guild appears in more than one aspect. Multi Hits occur when multiple artificial guilds appear together in a single aspect. Hit rate, Extra Hits, and Multi Hits are shown as percent values (%).*

	K=5			*K=10*			*K=20*		
Guild Size/ Abundance	Hit Rate	Extra Hits	Multi Hits	Hit Rate	Extra Hits	Multi Hits	Hit Rate	Extra Hits	Multi Hits
5/0.02	0	0	0	16.0	0	1.0	81.7	0	7.7
5/0.05	0	0	0	88.0	0	11.3	99.7	0.7	4.0
5/0.1	22.3	0	0	100	0	9.7	100	12.3	0
7/0.02	0	0	0	40.0	0	3.3	95.0	0	7.3
7/0.05	2.0	0	0	94.0	0	18.7	100	0	1.3
7/0.1	64.3	0	10.0	100	0	3.3	100	12.0	0
9/0.02	0	0	0	70.1	0	8.0	98.7	0	7.3
9/0.05	14.0	0	2.0	99.0	0	15.0	100	0	1.0
9/0.1	87.7	0	21.0	100	0	0.3	100	17.4	0

Supplemental Table 3: *Results of Aspect Bernoulli runs with three artificial guilds inserted randomly at three aspect numbers K=5,10,20. Hit Rate describes the percentage of hits observed out of all possible hits. Extra Hits represent runs where an artificial guild appears in more than one aspect. MultiHits occur when multiple artificial guilds appear together in a single aspect. Hit rate, Extra Hits, and Multi Hits are shown as percent values (%).*

	K=5			*K=10*			*K=20*		
Guild Size/ Abundance	Hit Rate	Extra Hits	Multi Hits	Hit Rate	Extra Hits	Multi Hits	Hit Rate	Extra Hits	Multi Hits
5/0.02	0	0	0	11.7	0	0	81.7	0.3	6.3
5/0.05	0	0	0	87.7	0	19.3	100	0.7	8.9
5/0.1	23.3	0	3.0	100	0	23.0	100	11.5	5.3
7/0.02	0	0	0	46.7	0	4.3	95.0	0	10.7
7/0.05	4.3	0	0.3	95.3	0	25.0	100	0.3	6.6
7/0.1	78.3	0	26.3	100	0	17.7	100	15.7	2.0
9/0.02	0	0	0	69.0	0	8.3	99.3	0	8.3
9/0.05	27.3	0	9.7	98.7	0	23.7	100	0.3	2.7
9/0.1	92.7	0	32.0	100	0	8.0	100	16.2	0.6

Supplemental Table 4: *Guilds defined as top 5 functions of each aspect for a run of the AB model on the composite dataset with K=10 aspects.*

Guild 1	CoenzymeB/CoenzymeMregeneration	Methanogenesisviaacetate	molybdate/tungstateSBP	dissimilatorysulfite<>sulfide	dissimilatorysulfite<>APS
Guild 2	TypeIISecretion	Ubiquinol-cytochromecreductase	Cytochromecoxidase,cbb3-type	Flagellum	phospholipidSBP
Guild 3	DMSPdemethylation	DMSPlyase(dddLQPDKW)	sulfitedehydrogenase(quinone)	Methanogenesisviatrimethylamine	dimethylamine/trimethylaminedehydrogenase
Guild 4	NAD(P)H-quinoneoxidoreductase	Cytochromeb6/fcomplex	PhotosystemII	PhotosystemI	putativechitobioseSBP
Guild 5	C-PlyasecleavagePhnJ	CP-lyaseoperon	CP-lyasecomplex	fructoseSBP	d-xyloseSBP
Guild 6	glutamateSBP	cellobioseSBP	arabinogalactanSBP	d-methionineSBP	n-diacetylchitobioseSBP
Guild 7	maltose/maltodextrinSBP	transporter:vitaminB12	arginineSBP	putativeaminoacidSBP	d-methionineSBP
Guild 8	sulfurassimilation	Retinalbiosynthesis	manganese/zinc/ironSBP	nitrous-oxidereduction	gluconeogenesis
Guild 9	V-typeATPase	NADH-quinoneoxidoreductase	glycolysis	riboflavinbiosynthesis	MethanogenesisviaCO_2
Guild 10	rhodopsin	beta-carotene15,15-monooxygenase	Retinalbiosynthesis	Mixedacid:Ethanol,AcetatetoAcetylaldehyde	Cytochromecoxidase

Supplemental Table 5: *Guilds defined as top 5 functions of each aspect for a run of the AB model on the MAG-only dataset with K=10 aspects.*

Guild 1	molybdate SBP	glycinebetaine/proline SBP	DMSP lyase (dddLQPDKW)	hydroxymethylpyrimidine SBP	fMethanogenesis via trimethylamine
Guild 2	Cytochrome-c oxidase cbb3 type	Type II secretion	Cytochrome bd complex	DNRA	Chemotaxis
Guild 3	gluconeogenesis	Gamma.hexachlorocyclohexane SBP	Type I secretion	Sec.SRP	Mixedacid.Ethanol.AcetatetoAcetylaldehyde
Guild 4	ribose SBP	manganes/zinc/iron SBP	sulfur assimilation	Mixedacid.Ethanol.Acetyl.CoAtoAcetylaldehyde.reversible	Entner.Doudoroff Pathway
Guild 5	DMSP demethylation	putative spermidine/putrescine SBP	sulfite dehydrogenase (quinone)	dimethylamine/trimethylamine dehydrogenase	thiosulfate oxidation
Guild 6	Mixedacid.Acetate	glycolysis	F-type ATPase	putative multiple sugar SBP	peptides/nickel SBP
Guild 7	V-type ATPase	NADH-quinone oxidoreductase	Mixedacid-PEP to Succinate via OAA/malate/fumarate	rhodopsin	Anaplerotic genes
Guild 8	C-P lyase cleavage PhnJ	CP-lyase operon	CP-lyase complex	d-methionine SBP	trehalose/maltose SBP
Guild 9	ubiquinol cytochrome-c reductase	Type I Secretion	rhodopsin	beta.carotene15.15.monooxygenase	ammonia transporter
Guild 10	Mixedacid.Formate	Cobal/nickel SBP	Methanogenesis via acetate	CoenzymeB/CoenzymeM regeneration	rhamnose SBP

Supplemental Table 6: *Guilds defined as top 5 functions of each aspect for a run of the AB model on the SAG-only dataset with K=10 aspects.*

Guild 1	NAD(P)H-quinone oxidoreductase	Cytochrome b6/f complex	Photosystem I	Photosystem II	manganese/zinc SBP
Guild 2	zinc SBP	biotin SBP	DMSP demethylation	taurine SBP	general l-amino acid SBP
Guild 3	dissimilatory sulfite<>APS	DMSP demethylation	biotin SBP	NADP-reducing hydrogenase	branched chain amino acid SBP
Guild 4	Ubiquinol-cytochrome c reductase	Type II Secretion	Naphthalene degradation to salicylate	Gyloxylate shunt	Type I Secretion
Guild 5	CP-lyase operon	CP-lyase complex	C-P lyase cleavage PhnJ	transporter: phosphonate	glucose/mannose SBP
Guild 6	Competence-related core components	iron complex SBP	dipeptide SBP	glutamine SBP	riboflavin biosynthesis
Guild 7	gluconeogenesis	anoxygenic type-II reaction center	cystine SBP	Mixed acid: Formate to CO2 & H2	Methanogenesis via trimethylamine
Guild 8	putative simple sugar SBP	trehalose/maltose SBP	putative sn-Glycerol 3-phosphate SBP	glutathione SBP	ribose SBP
Guild 9	Methanogenesis via CO2	cobalamin biosynthesis	sulfur assimilation	V-type ATPase	gamma-hexchlorocyclohexane SBP
Guild 10	raffinose/stachyose/melibioseSBP	peptides/nickel SBP	putrescine SBP	arginine SBP	arabinosaccharide SBP

Supplemental Table 7: *Number of mapback genomes for guilds defined by the top 5 highest scoring functions for three different values of K (K=5,10,20). X's represent guilds beyond the size of K.*

Guild	Number of Mapbacks (*K=5)*	Number of Mapbacks (*K=10*)	Number of Mapbacks (*K=20*)
Guild 1	109	11	51
Guild 2	65	468	12
Guild 3	1295	68	21
Guild 4	282	45	171
Guild 5	61	97	210
Guild 6	X	49	1
Guild 7	X	62	51
Guild 8	X	37	69
Guild 9	X	90	8
Guild 10	X	235	24
Guild 11	X	X	14
Guild 12	X	X	49
Guild 13	X	X	97
Guild 14	X	X	50
Guild 15	X	X	0
Guild 16	X	X	150
Guild 17	X	X	46
Guild 18	X	X	11
Guild 19	X	X	9
Guild 20	X	X	46

Supplemental Table 8: *Guilds for the two models generated to assess the numerical stability of the AB procedure. Each column reflects the functions from one model with the rows distinguishing which guild they belonged to. For visual ease, a blank row is inserted between guilds.*

Guilds	Model 1	Model 2
Guild 1	glutamate SBP	glutamate SBP
Guild 1	arabinogalactan SBP	arabinogalactan SBP
Guild 2	arginine/ornithine SBP	arginine/ornithine SBP
Guild 2	maltose/maltodextrin SBP	maltose/maltodextrin SBP
Guild 3	beta-carotene15,15-monoxygenase	beta-carotene15,15-monoxygenase
Guild 3	Retinal biosynthesis	Retinal biosynthesis
Guild 3	Type I Secretion	Type I Secretion
Guild 3	gamma/hexachlorocyclohexane SBP	gamma/hexachlorocyclohexane SBP
Guild 3	gluconeogenesis	gluconeogenesis
Guild 4	sulfite dehydrogenase (quinone)	sulfite dehydrogenase (quinone)
Guild 4	DMSP lyase (dddLQPDKW)	DMSP lyase (dddLQPDKW)
Guild 4	DMSP demethylation	DMSP demethylation
Guild 5	glycolysis	glycolysis
Guild 5	NADH-quinone oxidoreductase	NADH-quinone oxidoreductase
Guild 5	riboflavin biosynthesis	riboflavin biosynthesis
Guild 5	Anaplerotic genes	V-type ATPase
Guild 5	F-type ATPase	Anaplerotic genes
Guild 6	Ubiquinol-cytochrome c reductase	Ubiquinol-cytochrome c reductase
Guild 6	Type II Secretion	Type II Secretion
Guild 6	Cytochrome c oxidase cbb3-type	Cytochrome c oxidase cbb3-type
Guild 6	phospholipid SBP	phospholipid SBP
Guild 6	Flagellum	Flagellum

1. Xu, S. *et al.* Use ggbreak to Effectively Utilize Plotting Space to Deal With Large Datasets and Outliers. *Front. Genet.* **12**, 774846 (2021).
2. Galili, T. dendextend: an R package for visualizing, adjusting and comparing trees of hierarchical clustering. *Bioinformatics* **31**, 3718–3720 (2015).
3. Bingham, E., Kabán, A. & Fortelius, M. The aspect Bernoulli model: multiple causes of presences and absences. *Pattern Anal. Appl.* **12**, 55–78 (2009).
4. Kuhn, H. W. The Hungarian method for the assignment problem. *Nav. Res. Logist. Q.* **2**, 83–97 (1955).
5. Graham, E. D., Heidelberg, J. F. & Tully, B. J. Potential for primary productivity in a globally-distributed bacterial phototroph. *ISME J.* **12**, 1861–1866 (2018).
